# SARS-CoV-2 infection in hamsters and humans results in lasting and unique systemic perturbations post recovery

**DOI:** 10.1126/scitranslmed.abq3059

**Published:** 2022-06-07

**Authors:** Justin J. Frere, Randal A. Serafini, Kerri D. Pryce, Marianna Zazhytska, Kohei Oishi, Ilona Golynker, Maryline Panis, Jeffrey Zimering, Shu Horiuchi, Daisy A. Hoagland, Rasmus Møller, Anne Ruiz, Albana Kodra, Jonathan B. Overdevest, Peter D. Canoll, Alain C. Borczuk, Vasuretha Chandar, Yaron Bram, Robert Schwartz, Stavros Lomvardas, Venetia Zachariou, Benjamin R. tenOever

**Affiliations:** ^1^ Department of Microbiology, Icahn School of Medicine at Mount Sinai, New York, NY 10029; ^2^ Department of Microbiology, New York University, Grossman School of Medicine, New York, NY 10016; ^3^ Department of Neuroscience, Icahn School of Medicine at Mount Sinai, New York, NY 10029; ^4^ Mortimer B. Zuckerman Mind, Brain and Behavior Institute, Columbia University, New York, NY 10027; ^5^ Department of Neurosurgery, Icahn School of Medicine at Mount Sinai, New York, NY 10029; ^6^ Department of Immunology, Harvard Medical School, Boston, MA 02115; ^7^ Department of Otolaryngology- Head and Neck Surgery, Columbia University Irving Medical Center, Vagelos College of Physicians and Surgeons, Columbia University, New York, NY 10032; ^8^ Department of Pathology and Cell Biology, Columbia University Irving Medical Center, Vagelos College of Physicians and Surgeons, Columbia University, New York, NY 10032; ^9^ Department of Pathology and Laboratory Medicine, Weill Cornell Medicine, New York, NY 10021; ^10^ Department of Physiology, Biophysics, and Systems Biology, Weill Cornell Medicine, New York, NY 10021; ^11^ Division of Gastroenterology and Hepatology, Department of Medicine, Weill Cornell Medicine, New York, NY 10021

## Abstract

The host response to severe acute respiratory syndrome coronavirus 2 (SARS-CoV-2) infection can result in prolonged pathologies collectively referred to as post-acute sequalae of COVID-19 (PASC) or long COVID. To better understand the mechanism underlying long COVID biology, we compared the short- and long-term systemic responses in the golden hamster following either SARS-CoV-2 or influenza A virus (IAV) infection. Results demonstrated that SARS-CoV-2 exceeded IAV in its capacity to cause permanent injury to the lung and kidney and uniquely impacted the olfactory bulb (OB) and epithelium (OE). Despite a lack of detectable infectious virus, the OB and OE demonstrated myeloid and T cell activation, proinflammatory cytokine production, and an interferon response that correlated with behavioral changes extending a month post viral clearance. These sustained transcriptional changes could also be corroborated from tissue isolated from individuals who recovered from COVID-19. These data highlight a molecular mechanism for persistent COVID-19 symptomology and provide a small animal model to explore future therapeutics.

## Teaser

SARS-CoV-2 infection results in sustained inflammation in the nervous system and is a driver of long COVID.

## INTRODUCTION

Severe acute respiratory syndrome coronavirus 2 (SARS-CoV-2) is a respiratory RNA virus that emerged in 2019 and is associated with a variety of clinical phenotypes ranging from asymptomatic to more severe disease generally referred to as coronavirus disease (COVID-19) (*1*). In most cases among young and healthy individuals, COVID-19 is characterized by a relatively mild flu-like illness and includes limited respiratory tract congestion, fever, myalgia, headache, and anosmia (*2*–*4*). Amongst older populations, especially males and those with co-morbidities, COVID-19 can result in severe respiratory distress, multi-organ complications, and death (*1*, *5*).

Regardless of age or underlying health, virus infection is thought to impair host transcriptional and translational processes to enhance replication (*6*–*8*). As a result, infected cells are unable to elicit a Type I interferon (IFN-I) response, a central mediator of the host’s antiviral defenses through the up-regulation of hundreds of antiviral interferon-stimulated genes (ISGs) (*9*, *10*). During a SARS-CoV-2 infection, induction of IFN-I largely derives from uninfected cells, such as resident macrophages and other phagocytic cells (*11*). Despite blocking many aspects of the host antiviral response, SARS-CoV-2 infection relies on persistent signaling of the nuclear factor κB (NFκB) transcription factor family, indirectly culminating in transcriptional activation of proinflammatory cytokines and chemokines such as interleukin (IL)-6 and CXCL10, respectively (*9*, *12*, *13*). As a result of these dynamics, neutrophils and monocytes amass in the respiratory tract as the virus propagates in an environment with suboptimal antiviral defense engagement - further exacerbating the inflammatory environment. Virus infection results in extensive damage to the bronchial epithelium and pulmonary edema, ultimately leading to a loss of normal lung function (*2*–*4*).

Characterization of SARS-CoV-2 biology has identified angiotensin converting enzyme 2 (ACE2) and a subset of proteases that enable viral entry (*14*–*16*). Despite the expression of these host factors on multiple tissues, productive SARS-CoV-2 infection appears to be largely contained in the respiratory tract (*2*–*4*). Selective localization in the airways, however, is not a product of viral tropism; rather, it is a byproduct of the systemic IFN-I response that initiates at the site of infection, enabling distal tissues to become recalcitrant to subsequent infection. For example, human organoid models have demonstrated productive infection of diverse tissues ex vivo despite being rarely observed in vivo (*17*–*19*). This phenomenon can also be modeled in the golden hamster, one of the most widely used small animal models for COVID-19, which demonstrates consistent infection of the respiratory tract and olfactory epithelium upon intranasal challenge, with only sporadic isolation of virus from other tissues unless IFN-I biology is disrupted (*20*–*24*). This finding is further supported by the fact that SARS-CoV-2 has been seen to readily infect ex vivo organotypic cultures of hamster brain tissues, which are not readily seen to be sites of viral replication in intranasal infection conditions (*21*, *25*). This same phenomenon can be observed when infected individuals are immunosuppressed (*17*, *26*–*28*). Although it remains unclear how common infection of distal tissues occurs during a SARS-CoV-2 infection, system-wide inflammation is consistent (*2*–*4*). Together these data suggest that the molecular underpinnings of acute COVID-19 are a by-product of the damage caused by the virus and the systemic response that ensues.

In most individuals, virus infection is successfully cleared with the appearance of neutralizing antibodies to the spike attachment protein. Generally, the appearance of the humoral response correlates to resolution of the symptoms associated with SARS-CoV-2 (*29*–*31*). However, a growing body of evidence suggests that, in a subset of individuals, SARS-CoV-2 infection results in prolonged complications including shortness of breath, persistent fevers, fatigue, depression, anxiety, and a state of chronic impairment of memory and concentration known colloquially as “brain fog”. The direct cause of these impairments, known collectively as “long COVID” or post-acute sequalae of COVID-19 (PASC), is currently unknown (*32*, *33*).

To better understand the prolonged effects caused by SARS-CoV-2 infection, we focused on the golden hamster as a model system. The hamster model has proven to largely phenocopy COVID-19 biology without any requirement for SARS-CoV-2 adaptation and has demonstrated a propensity to display severe lung morphology and a tropism that matches what is observed in human patients (*20*, *21*, *23*, *34*). In these studies, we characterize the host response to SARS-CoV-2 and benchmark the findings to a former Influenza A Virus (IAV) pandemic virus infection (*35*).

Here we show that although both IAV and SARS-CoV-2 induce a systemic antiviral response, only the latter infection results in a sustained inflammatory pathology that extends well beyond clearance of the primary infection. As this sustained inflammation also correlates with behavioral abnormalities, we propose that this biology may underlie the cause of long COVID in both humans and hamsters.

## RESULTS

### SARS-CoV-2- and IAV-infected hamsters develop a host response that mirrors human biology and resolves within two weeks post infection.

**Fig. 1.**
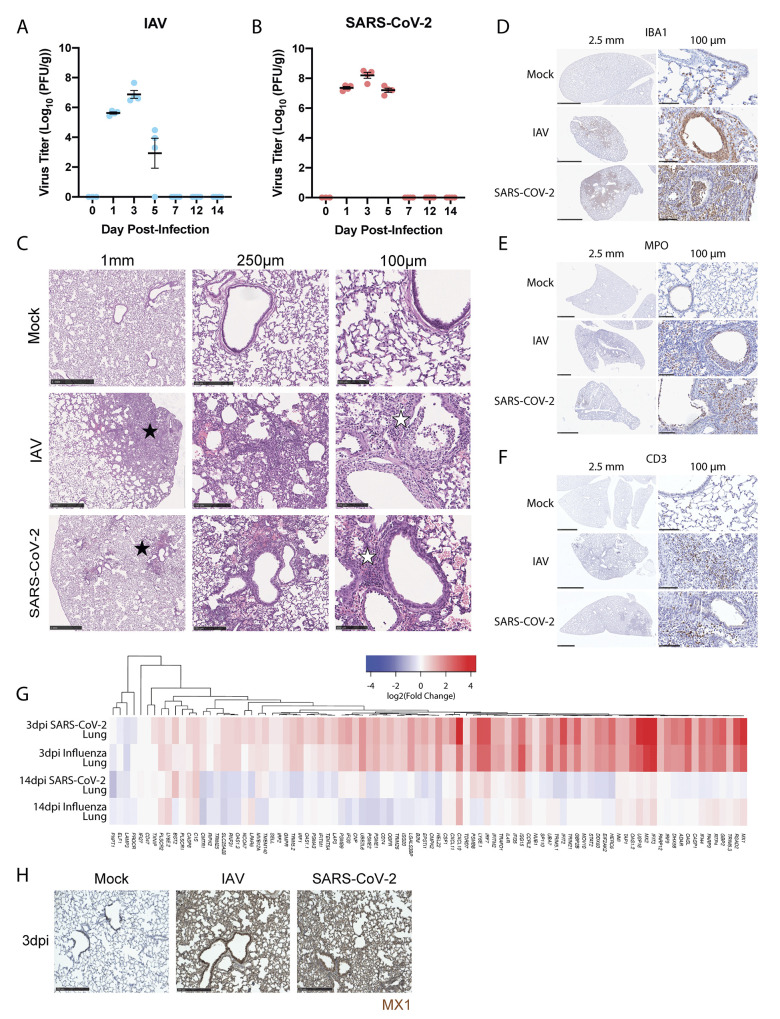
**SARS-CoV-2 and IAV infections induce clinically representative lung pathology and are cleared by 14dpi in the hamster model of disease. (A and B)** Titer data was computed as plaque forming units per gram (PFU/g) of lung from hamsters infected with **(A)** IAV (A/California/04/2009) (n=4 per time point) or **(B)** SARS-CoV-2 (USA/WA1/2020) (n=4 per time point) on days indicated. Day 0 is representative of uninfected hamsters (n=3). **(C)** H&E staining of hamster lungs exposed to PBS (Mock), IAV, or SARS-CoV-2 at 3dpi is shown. Histological analysis at various magnifications denoting hypercellularity (black star) and infiltrate presence in bronchioles and alveoli (white star). Scale bar size is denoted above the images. **(D to F)** Immunohistochemical labeling for **(D)** IBA1, **(E)** MPO, and **(F)** CD3 were used to label macrophage, neutrophil, and T cell populations, respectively, in the lungs of mock-, IAV-, and SARS-CoV-2-infected hamsters at 3dpi. Size of inset scale bars matches length described in column headers. **(G)** RNA-seq of lungs from SARS-CoV-2- and IAV-infected hamsters was evaluated at 3dpi and 14dpi. Heatmap depicting log2 fold-change of IFN-I response genes (derived from HALLMARK_INTERFERON_ALPHA_RESPONSE gene set) compared to mock-infected animals was generated for these groups. **(H)** Immunohistochemical labeling for the interferon stimulated gene MX1 was assessed in lungs of mock-infected, IAV-infected, or SARS-CoV-2-infected hamsters at 3dpi. Scale bars indicate 250μm length.

To define the unique characteristics of SARS-CoV-2 infection that may contribute to persistent symptomology, we performed a longitudinal study in hamsters infected with either SARS-CoV-2 [USA-WA1/2020, 10^3^ plaque-forming units (pfu)] or IAV (pandemic H1N1 isolate A/California/04/2009, 10^5^ pfu). Inoculation dosages were informed by past studies to achieve comparable kinetics and viral load in these two model systems (*21*, *36*). Longitudinal study data demonstrated that both respiratory RNA viruses could replicate in the lungs of the golden hamster, albeit showing a modest difference with regards to clearance, consistent with what has been reported elsewhere (Fig. 1A and B, fig. S1A and B) (*21*, *37*). The delayed clearance of SARS-CoV-2 further coincided with a diminished appetite, as this cohort of hamsters gained weight more slowly than either the phosphate-buffered saline (PBS)-treated or IAV infected animals, as we have reported elsewhere (36). IAV challenge resulted in peak titers of 10^7^ pfu per gram of lung tissue (pfu/g) on day three followed by a sharp decline in infectious material, showing a complete loss of infectivity by 7 days post-infection (dpi) (Fig. 1A). For SARS-CoV-2, we also observed peak viral titers at 3dpi (about 10^8^ pfu/g), which persisted until day 5 before declining (Fig. 1B). Despite the difference in sustained virus replication upon reaching peak virus titers in both model systems, no infectious virus could be isolated on day 7; however, RNA remained detectable through quantitative reverse-transcription-based PCR (qRT-PCR) for the nucleoprotein of influenza (NP) as well as the sub-genomic RNA of nucleocapsid (sgN) from SARS-CoV-2 (Fig. 1A and B and fig. S1A and B). Based on these data, we focused on day 3 to compare the acute host response to these two respiratory infections, as this time point appeared to be the most comparable between the two viruses with regards to kinetics and viral load during the disease course. Furthermore, as 3dpi corresponded to peak infection for both viruses, we hypothesized that this time point would likely be the point in which pathologies attributable to direct viral activity would be most visible.

To compare the pathology resulting from IAV versus SARS-CoV-2, we examined cross sections of the hamster lung at 3dpi using various histological techniques that were evaluated by a board-certified pathologist (Fig. 1C to F and fig. S1C). Hematoxylin and eosin (H&E) treatment of lung tissue derived from hamsters infected with either IAV or SARS-CoV-2 revealed large areas of intense staining (Fig. 1C, black stars, and fig. S1C) that, at higher magnification, implicated hypercellularity and the infiltration of inflammatory cells into both alveolar compartments and bronchiolar airway spaces (white stars). To better characterize the cellular content of the pulmonary inflammatory infiltrate, immunohistochemical (IHC) staining was used to label macrophages, which express Ionized calcium binding adaptor molecule 1 (IBA1) (Fig. 1D), neutrophils, which express myeloperoxidase (MPO) (Fig. 1E), and T cells, which express CD3 (Fig. 1F) on these same cross-sections. These efforts demonstrated that the high hematoxylin-stained regions of the lung, regardless of virus, showed extensive positivity for all three cell types, with neutrophils and macrophages predominating (Fig. 1C to F). One notable difference between these virus models was that SARS-CoV-2 induced pulmonary infiltration that was centrally located around bronchioles and larger airway structures (Fig. 1C, black star).

In contrast to the lung, examination of distal tissues, including kidney and heart, showed moderate to no pathological features at 3dpi for either virus (fig. S1D and E). We observed no signs of cellular infiltration in the kidney at this time point, whereas in the heart, an organ often associated with COVID-19 complications (*38*), we noted some evidence for inflammation and leukocytic infiltrate in response to both viruses (fig. S1E, green stars). Together, these data suggest that the hamster phenocopies many of the histological characteristics seen in the human response to IAV or SARS-CoV-2 during acute infection (*4*).

To characterize the molecular dynamics of these model systems, we next performed RNA sequencing (RNA-seq) on infected lungs isolated at 3dpi (Fig. 1G and fig. S1F and G). These data identified about 100 differentially expressed genes (DEGs) with a P-adjusted (p-adj) value of less than 0.1 in both SARS-CoV-2- and IAV-infected lungs. Gene set enrichment analyses (GSEA) against hallmark gene ontology sets implicated activation of the IFN-I, IFN-II, tumor necrosis factor (TNF)-α, and IL-2 signaling pathways in response to either infection (fig. S1H). These data could be further corroborated by immunohistochemistry of fixed lung tissue probed for the ISG MX1 (Fig. 1H). We next examined the transcriptional response to SARS-CoV-2- and IAV-infected hamsters at 14dpi, about 1 week post clearance (Fig. 1A, B, and G). In contrast to the inflammation observed at 3dpi, sequencing SARS-CoV-2- or IAV-infected lung tissue at 14dpi showed minimal signs of an antiviral response (Fig. 1G). Together, these data demonstrate that the golden hamster model shows a robust acute response in the respiratory tract that successfully resolves both IAV or SARS-CoV-2 infection.

### Transcriptional profiling of peripheral organs reveals differences between active or resolved IAV and SARS-CoV-2 infections.

**Fig. 2.**
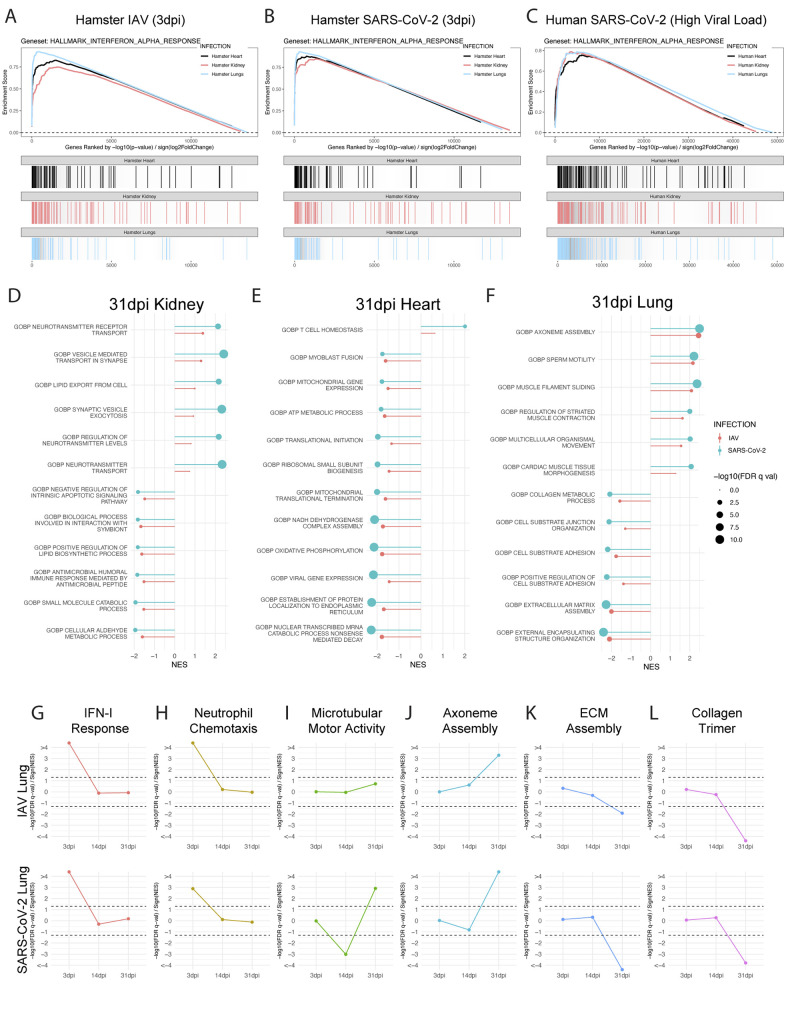
**Transcriptional profiling of peripheral organs reveals differences between active or resolved IAV and SARS-CoV-2 infections. (A to C)** Lungs (blue), kidneys (red), and hearts (black) of SARS-CoV-2-, IAV-, and mock-treated hamsters were harvested at 3dpi and transcriptionally profiled by RNA-seq. Differential expression analysis was conducted between infected and mock-infected groups with DESeq2 and analyzed by Gene Set Enrichment Analysis (GSEA) for enrichment of indicated gene sets. Enrichment analysis results for all three tissue types are displayed in a GSEA enrichment plot for **(A)** IAV versus Mock and **(B)** SARS-CoV-2 versus Mock comparisons. **(C)** Similar transcriptomic analyses were conducted on RNA-seq data generated from human lung, heart, and kidney samples obtained from the post-mortem tissues of COVID-19-infected and control donors. Results from enrichment analyses are shown as a GSEA enrichment plot. **(D to F)** Differential expression analysis of RNA-seq data derived from **(D)** lungs, **(E)** kidneys, and **(F)** hearts of SARS-CoV-2-, IAV-, and mock-infected hamsters at 31dpi is shown. Differential expression results were assessed using GSEA to test for enrichment of gene sets present in the MSigDB C5 gene set collection, which contains curated gene sets derived from the Gene Ontology resource. Significant ontological enrichments for SARS-CoV-2 versus Mock differential expression analysis were further processed by REVIGO to remove redundant enrichments. The highest ranked non-redundant positive and negative enrichments for each organ are plotted by their normalized enrichment score (NES) (line magnitude) and significance (-log10(FDR q-value)) (dot size). GSEA enrichment for these same gene sets in IAV versus Mock differential expression data for the same tissue are plotted side-by-side for comparison. (Gene Ontology Biological Process: GOBP) **(G to L)** GSEA analysis from lung sequencing data from IAV-, SARS-CoV-2, and mock-infected hamster lungs at 3, 14, and 31dpi is shown using curated gene ontology and human phenotype ontology gene sets. Directional significance of enrichment was plotted over time for (**G**) IFN-I response (GOBP_RESPONSE_TO_TYPE_I_INTERFERON) (**H)** neutrophil chemotaxis (GOBP_NEUTROPHIL_CHEMOTAXIS) (**I**) microtubular motor activity (GOMF_ATP_DEPENDENT_MICROTUBULE_MOTOR_ACTIVITY) (**J**) axoneme assembly (GOBP_AXONEME_ASSEMBLY) (**K**) extracellular matrix (ECM) assembly (GOBP_EXTRACELLULAR_MATRIX_ASSEMBLY) (**L**) and collagen trimer-associated genes (GOCC_COLLAGEN_TRIMER). Dotted lines show the calculated statistic for FDR q-val = 0.05 for positive and negative enrichment; thus, any points falling outside the dotted lines have FDR q-val of < 0.05. (GOMF: Gene Ontology Molecular Function; GOCC: Gene Ontology Cellular Component)

To corroborate the clinical validity of the SARS-CoV-2 acute hamster data, we compared our RNA-seq analyses to published results from lungs of COVID-19 deceased individuals that still had high viral loads at the time of death (Fig. 2A to C) (*39*). In agreement with the published data, we found transcriptional signatures from both groups were dominated by a marked up-regulation of the IFN-I and TNF-α signaling pathways (*9*, *13*, *21*). As the host response to acute IAV and SARS-CoV-2 were comparable in the respiratory tract, we next sought to characterize distal tissues and expand our characterization to time points representing both active and resolved infections. Although COVID-19 symptoms usually resolve within four weeks post infection onset, symptoms can persist much longer in a subset of patients. Patients demonstrating symptoms lasting longer that four weeks following infection have now been clinically defined as having long COVID or PASC (*40*). To this end, we conducted additional transcriptional profiling on the lung (blue), heart (black), and kidneys (red) from hamsters infected with SARS-CoV-2 or IAV at 3dpi and 31dpi, the latter being a timepoint where any symptom-generating pathology would be clinically defined as long COVID in a human patient (Fig. 2A and B and fig. S2A to C).These data were also cross-referenced to matching tissues derived from human cadaver specimens that were infected with COVID-19 at the time of death, as described elsewhere (Fig. 2C) (*41*). These comparisons encompassed more than 50 samples at both early and late time points, which clustered based on tissues from which they derived (fig. S2D).

To first assess the acute response in a more systemic fashion, we utilized GSEA to characterize curated ontology gene sets from the aforementioned tissues. These efforts implicated a strong acute induction of the IFN-I response [false-discovery rate (FDR) q-val < 0.0001] in all three organs following either SARS-CoV-2 or IAV infection that were also evident in corresponding human tissues (Fig. 2A to C, fig. S2E to G). Also of note, IFN-I signatures in the lung of hamsters and COVID-19 cadavers were also accompanied by up-regulation of IFN-I-associated pathways, including NFκB- and IL-6-associated target genes (fig. S2H to J). Other enriched pathways induced by SARS-CoV-2 or IAV infection included positive regulation of complement activation in the kidney and negative regulation of calcium channel formation in the heart, although these enrichments were relatively minor in comparison to the IFN-I signatures (fig. S2E to H). Together these data corroborate independent published studies and provide further support for the use of the golden hamster as a model for acute SARS-CoV-2 pathology (*20*, *21*).

Having established the hamster as a clinical proxy for systemic acute pathology in response to a respiratory infection, we next sought to extrapolate these findings to any possible long-term consequences. To this end we performed similar analysis on lung, heart, and kidney tissues collected from hamsters at 31dpi, representing a time point greater than two weeks past disease resolution of the lungs (Fig. 1A and B, Fig. 2D to F). In agreement with the clearance of virus, these analyses failed to show any enrichment of IFN-I- or chemokine-related signatures in any of the tissues examined (Fig. 2D to F). Instead, tissue-specific annotations identified various biological processes involved in kidney resorption capacities and heart metabolism (Fig. 2D and E). In the lung, GSEA at 31dpi implicated general pathways of repair and regeneration (Fig. 2F). Amongst these was the biogenesis of cilia and airway repair in the lung following infection, which drives the up-regulation of genes involved in axoneme assembly, and filament sliding, which are also involved in pathways that were identified as 'sperm motility' (Fig. 2F).

To further visualize the development of the respiratory ontologies over time, we combined all the sequencing performed on days 3, 14, and 31dpi and mapped their significance values (Fig. 2G to L). These data supported our earlier findings that SARS-CoV-2 and IAV infections were resolved by day 14, as the IFN-I response and neutrophil chemotaxis induced by these viruses showed a lack of enrichment at both days 14 and 31 post-infection, despite their strong induction at 3dpi (Fig. 2G and H).

Next, we assessed microtubular motor activity and ciliary assembly ontologies over this longitudinal comparison to better understand the dynamics of bronchiolar repair, given that this biology was enriched in both viral infection models at 31dpi (Fig. 2F). A longitudinal query for ciliary-related ontologies showed no enrichment of these same transcripts during the acute phase of the two infection models, but a disparity between the cohorts one week post clearance (14dpi, Fig. 2I to J). At this time point, SARS-CoV-2-infected lungs uniquely displayed a negative enrichment of microtubular motor activity and a negative enrichment of axoneme assembly ontologies. As ciliary loss is part of the acute lung pathology following respiratory virus infection (*21*), we found the decline of microtubules and axoneme assembly-related genes specifically in response to SARS-CoV-2 noteworthy. These findings suggest that SARS-CoV-2-induced transcriptional aberrations may still be prevalent past 14dpi, even in the absence of infectious virus or proinflammatory transcriptional profiles. These data suggest that SARS-CoV-2-induced damage may be more severe and persist for a longer duration in the respiratory tract as compared to IAV. However, by 31dpi, the increase in microtubular motor activity and axoneme assembly likely reflect active regeneration of the ciliary machinery. This biology also corresponds with the down-regulation of genes associated with extracellular matrix assembly and collagen-trimer-related genes, which are involved in tissue regeneration (Fig. 2K to L) (*42*–*44*). The shared trends observed for the GSEAs suggests a resolving repair response at 31dpi following either IAV or SARS-CoV-2 infection.

### Histological characterization of lung, heart, and kidney tissue reveals differences in response to SARS-CoV-2 and IAV infection at 31dpi.

**Fig. 3.**
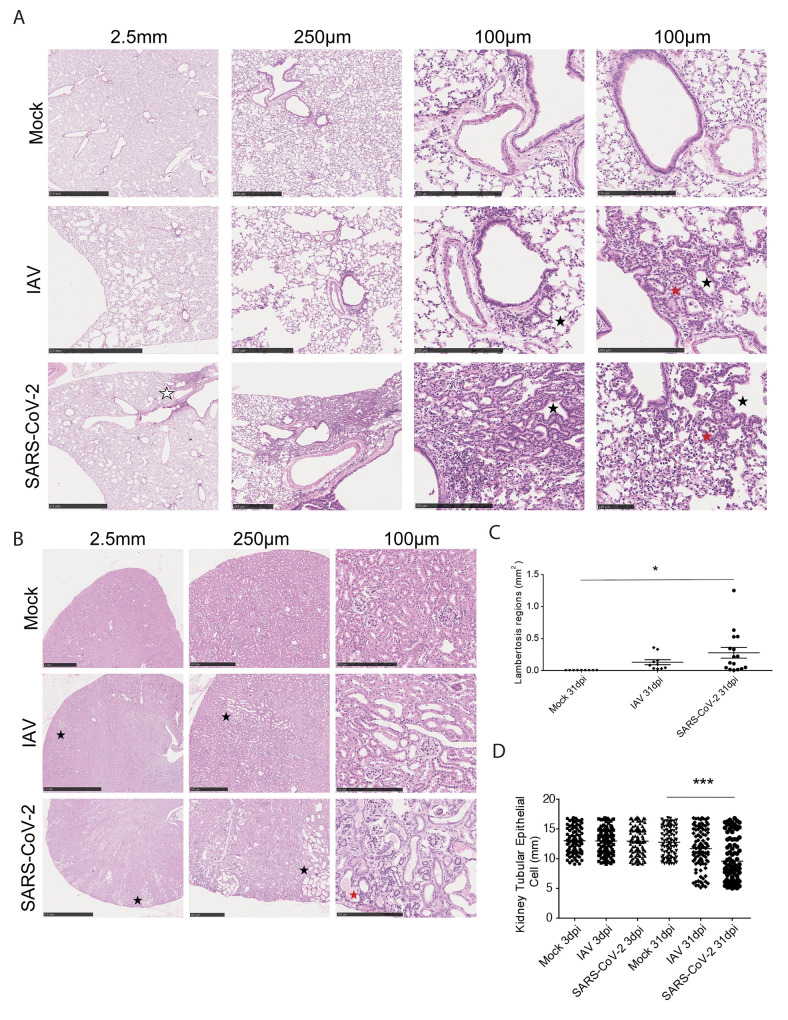
**Morphological characterization of lung, heart, and kidney reveal differences in response to SARS-CoV-2 or IAV at 31dpi. (A)** H&E staining on lungs of SARS-CoV-2-, IAV-, and mock-infected hamsters at 31dpi is shown. Histological analysis of lungs highlights lambertosis at both low magnification (white stars) and at higher magnification (black stars) as well as residual immune infiltration into lung parenchyma (red stars). **(B)** H&E staining on kidneys collected from the same infection groups as (A) is shown. Black and red stars denote areas of tubular atrophy and proteinaceous fluid buildup, respectively. **(C)** Lambertosis and **(D)** average tubular epithelial size were quantified by morphometric image analysis. Error bars display standard error mean, and significance was quantified using one-way ANOVA with Tukey’s Multiple Comparison Test; *p<0.05, ***p<0.001. For (C), 9 Mock, 10 IAV, and 16 SARS-CoV-2 randomly subsampled images of 31dpi lung tissues were analyzed from lung slides generated from 5 hamsters per treatment condition. For (D), 100 Mock, 100 IAV, and 100 SARS-CoV-2 randomly subsampled images of 3dpi and 31dpi kidney tissues were analyzed from kidney slides generated from 10 hamsters per treatment condition.

To assess long term organ damage independent of the transcriptional response, we profiled lung, heart, and kidney by histological analyses following IAV or SARS-CoV-2 infection at 31dpi (Fig. 3 and fig. S3). H&E staining revealed that both SARS-CoV-2- and IAV-infected lungs maintained their general structure, but displayed numerous abnormalities. Most prominent amongst these pathologies was peribronchiolar metaplasia (also known as lambertosis) (black stars), a clinical finding in which alveolar epithelial cells undergo metaplastic transformation to become bronchiolar-epithelium-like in appearance (Fig. 3A). This process generally occurs in response to severe respiratory trauma and can result in functional respiratory defects (*45*–*47*). In SARS-CoV-2-infected lungs, peribronchiolar metaplasia was more expansive and was visible even at minimal magnification when compared to the comparable IAV samples (white star) (Fig. 3A). Furthermore, lungs infected by both viruses showed signs of enlarged airway spaces and residual inflammation characterized by monocytes and neutrophils visible in the alveolar spaces (red stars, Fig. 3A). This residual inflammation is in agreement with our transcriptional profiling data, which found that *Cd177* and *Ly6d*, neutrophil- and monocyte-associated genes, respectively, were up-regulated in lungs infected by SARS-CoV-2 at 31dpi (fig. S2A). To confirm these findings, IHC staining was performed to label macrophage (IBA1), neutrophil (MPO), or T cell (CD3) populations in histological sections of lungs of infected hamsters at 31dpi (fig, S3A to C). In contrast to lungs from mock-infected animals, lungs from IAV- and SARS-CoV-2-infected animals showed localized areas of hypercellularity that stained positively for both neutrophil and macrophage populations (fig. S3A to C). Intriguingly, these hypercellular areas were commonly associated with areas of lambertosis, which could be distinguished by thickened alveolar walls compared to surrounding healthy and mock alveolar tissues. Additionally, in line with our sequencing, which identified a moderate and resolving repair response at 31dpi, Verhoeff Van Gieson staining, which labels collagen and elastin fibers, showed no obvious signs of fibrotic activity, collagen deposition, or elastin degradation in response to either infection (fig. S3D).

Given the persistent gene signatures on day 31 post-SARS-CoV-2 infection and the histological changes observed in the lung, we next assessed the kidney and heart by H&E staining (Fig. 3B and fig. S3D). In the heart, we observed complete resolution of leukocytic infiltration at 31dpi with no noteworthy histological signatures in response to infection (fig. S3E). In the kidney, however, SARS-CoV-2-infected animals displayed areas of tubular atrophy characterized by thinning of tubular cells and widening of the tubular lumen (black stars, Fig. 3B). Closer examination also revealed the presence of proteinaceous fluid in the interstitial space surrounding these tissues (red stars). Examination of the kidneys at this time point from IAV-infected hamsters showed similar pathological findings (black stars); however, the affected areas appeared smaller and less numerous than in SARS-CoV-2-infected hamsters, consistent with the notion that IAV-induced damage is less severe than that of SARS-CoV-2 in this small animal model.

To better assess the extent of infection-induced scarring, we performed quantitative morphometric analyses on these histological images. Quantification of lambertosis and airway size showed that these pathologies were greater in the lungs of SARS-CoV-2-infected animals (Fig. 3C and fig. S3F). A similar finding was also visible with respect to tubular atrophy and prior SARS-CoV-2 infection (Fig. 3D). Together, these data demonstrate that both SARS-CoV-2 and IAV infections present similar histological signatures in the lungs and in other peripheral organs. However, despite comparable host responses, we observed a greater severity of scarring in SARS-CoV-2 infection, which, given its nature, may predispose infected individuals to greater functional defects post viral clearance.

### SARS-CoV-2 induces unique neural transcriptional profiles compared to IAV.

**Fig. 4.**
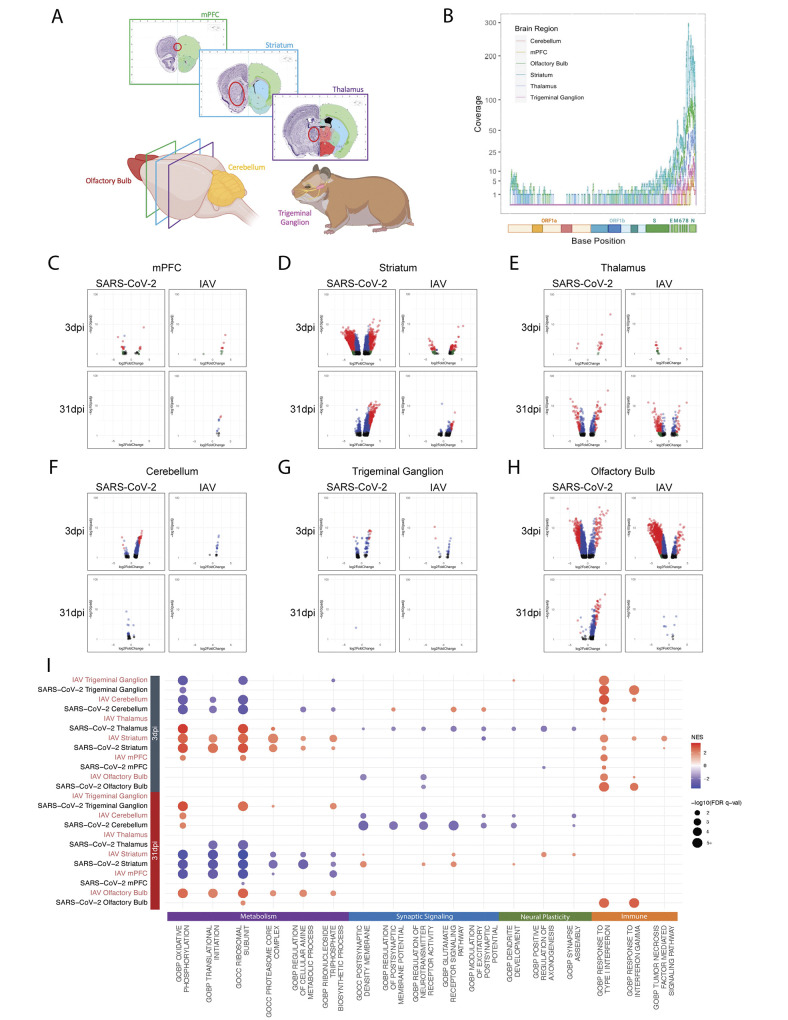
**SARS-CoV-2 induces a unique neural transcriptional profile compared to IAV. (A)** Schematic of brain regions characterized in response to infection. mPFC, striatum, thalamus, cerebellum, trigeminal ganglion, and olfactory bulb were bilaterally harvested for RNA-seq analysis. **(B)** Alignment of RNA-seq data to the SARS-CoV-2 genome is shown. Coverage of raw reads over the length of the genome are displayed as a histogram from each brain region noted. **(C to H)** Volcano plots depict differential expression analysis conducted using samples from the **(C)** mPFC, **(D)** striatum, **(E)** thalamus, **(F)** cerebellum, **(G)** trigeminal ganglion, and **(H)** olfactory bulb of IAV- or SARS-CoV-2-infected hamsters. Samples were compared to mock-infected hamsters at 3dpi and 31dpi using DESeq2; differentially expressed genes with a p-adjusted value of less than 0.1 are plotted (black: p-adj > 0.05, log2 fold-change < 2; blue: p-adj < 0.05, log2 fold-change < 2; green: p-adj > 0.05, log2 fold-change > 2; red: p-adj < 0.05, log2 fold-change > 2). **(I)** Differential expression data were analyzed by GSEA using curated gene ontology and human phenotype ontology gene sets; significant enrichments for metabolic-, synaptic signaling-, neural plasticity-, and immune-related ontologies are displayed on the dot plot. Coloration designates normalized enrichment score (NES), with positive enrichment scores colored red and negative enrichment scores colored blue. Dot size is scaled to -log10(FDR q-val) of enrichment; only enrichments with an FDR q-val of less than 0.05 were plotted.

Given that long COVID may also involve neurological and neuropsychiatric symptomology (*33*), we next assessed the consequences of SARS-CoV-2 infection on the nervous system. For these studies, we transcriptionally profiled several areas of the nervous system from 3 and 31dpi cohorts. More specifically, the areas surveyed included the olfactory bulbs, medial prefrontal cortex (mPFC), striatum, thalamus, cerebellum, and trigeminal ganglion (tissues collected as depicted in Fig. 4A). These areas were chosen either due to their previously documented positivity for SARS-CoV-2 transcripts in human patients (olfactory bulb, trigeminal ganglion) or due to their functional importance in sensory, motor, cognitive, or affective processes—all of which have been noted to be altered in subsets of patients with long COVID (*48*–*53*). Matched tissues from hamsters infected with IAV were also collected for comparison. Following tissue processing, brain regions from 3dpi were surveyed for the presence of viral RNA. As expected, in hamsters infected with IAV, no viral RNA could be detected from the surveyed neural tissue that aligned to the IAV genome (fig. S4A). In contrast, within the SARS-CoV-2-infected hamster cohort, viral reads were readily detectable in the nervous system in a subset of animals, consistent with the findings of others (*34*). Of note, in one hamster, SARS-CoV-2 reads were detectable in all surveyed regions of the nervous system (Fig. 4B). Mapping of these reads to the SARS-CoV-2 genome revealed that most reads aligned to the nucleocapsid (N) transcript, potentially implicating the deposition of circulating subgenomic RNA from a peripheral infection which is dominated by N (Fig. 4B) (*54*). Of note, these findings are consistent with reports of human patients displaying SARS-CoV-2 RNAemia and systemic detection of SARS-CoV-2 RNA that could reflect deposition of viral-derived inflammatory material (*26*, *55*).

To further characterize the appearance of SARS-CoV-2 genetic material in the brains of infected hamsters, a time course was conducted in which geographically distinct regions of the brain were sampled on days 1, 4, 7, and 14 post-infection in both SARS-CoV-2- and mock-infected hamsters. The olfactory bulb, striatum, and cerebellum were chosen for their respective positioning in the anterior, middle, and posterior sections of the cranial cavity. These regions were assessed for SARS-CoV-2 sgN transcripts by qRT-PCR and compared to lung, the primary site of infection (fig. S4B to E). Mirroring previous data (Fig. 1A and B, fig. S1A and B), SARS-CoV-2 sgN detection in the lungs was highest on days 1 and 4. By 7dpi, sgN detection diminished, with only negligible transcripts detectable at 14dpi (fig. S4B). In the olfactory bulb, a low abundance of sgN at 1dpi increased through 4dpi in two of three hamsters before dissipating over the next 7 days (fig. S4C). Moreover, striatum and cerebellum demonstrated different patterns of sgN positivity compared to both lungs and olfactory bulbs. At 1dpi, SARS-CoV-2-infected hamsters demonstrated sgN positivity in one out of three tested striatum sections and in all the cerebellum samples. Beyond this early time point, however, no cerebellum or striatum sections demonstrated sgN signal that rose above background (fig. S4D and E).

To assess whether sgN positivity was associated with triggering of an innate immune response, transcripts for *Isg15*, a canonical IFN-I stimulated gene, were assessed in sampled regions by qRT-PCR (fig. S4F to I). In general, *Isg15* signal correlated with sgN positivity; in lungs, for instance, *Isg15* transcripts were elevated on days 1, 4, and 7 post-infection, with its peak at 4dpi (fig. S4F). The striatum and cerebellum likewise show induction of *Isg15* signal at 1dpi, after which expression returned to baseline, mirroring the positive sgN signal in these same tissues (fig. S4D, E, G, and H). The olfactory bulbs show similar *Isg15* transcript abundance, following sgN signal on days 1, 4, and 7 post-infection; however, at 14dpi, the olfactory bulb intriguingly shows a newly elevated *Isg15* signal in the absence of any sgN positivity (fig. S4C and I).

To better understand the functional impacts that systemic SARS-CoV-2 and IAV challenge have on the nervous system, differential expression analyses of host transcripts were subsequently conducted across all sequenced neural areas from 3 and 31dpi following challenge with either SARS-CoV-2 or IAV and were compared to mock infection (Fig. 4C to H). To remain consistent to the prior studies, the time points chosen included 3dpi, representing peak infection, and a timepoint after viral clearance (31dpi), wherein no infectious material could be detected from the lungs (Fig. 1A and B). As RNA was isolated and sequenced for whole brain regions, differentially expressed genes observed in these analyses represent a global summation of transcriptional changes across all cellular populations present within the sampled tissue. Despite this limitation, several brain areas showed region-specific transcriptional alterations induced by viral infection at 3dpi. Intriguingly, this was evident for both SARS-CoV-2 and IAV (Fig. 4C to H). Direct comparison of SARS-CoV-2 and IAV by differential expression analyses revealed that most surveyed regions induced a very similar transcriptional profile between the two viruses at 3dpi (fig. S4J and K). These findings are most prominent in the striatum, where comparison of SARS-CoV-2 versus mock conditions revealed more than 3500 DEGs in contrast to the comparison of SARS-CoV-2 versus IAV, which demonstrated no significant transcriptional differences (Fig. 4D and fig. S4J). In contrast, these analyses also revealed different transcriptional profiles in many of the surveyed regions in response to SARS-CoV-2 and IAV infection at 3dpi. These differential responses to the two viral challenges were most prominent in the thalamus, cerebellum, and trigeminal ganglion (Fig. 4E to G and fig. S4J to K).

Additionally, this differential expression analyses demonstrated that transcriptional programs induced in neural tissue during viral challenge persisted for at least 1 month post-infection. Indeed, all surveyed regions showed DEGs in response to at least one of the viruses at 31dpi, albeit strikingly few changes observed in the trigeminal ganglion (Fig. 4C to H). These transcriptional signatures were comparable between SARS-CoV-2 and IAV in the striatum, mPFC, and cerebellum (fig. S4J to K). However, similar to the acute response at 3dpi, we again observed specific neural regions in which a unique transcriptional signature persists in response to SARS-CoV-2. These virus-specific signatures can be found in the striatum and olfactory bulb (Fig. 4D and H and fig. S4J to K).

To better understand the underlying biology responsible for the observed transcriptional changes taking place during infection, we again performed an unbiased GSEA. These efforts implicated four general host response signatures, including metabolism, synaptic signaling, neuronal plasticity, and immune activation (Fig. 4I). At 3dpi, widespread metabolic modulation encompassing changes to mitochondrial oxidative phosphorylation, protein translation, protein degradation, nucleotide synthesis, and amino acid synthesis pathways was observed within the surveyed neural tissues in response to both SARS-CoV-2 and IAV (Fig. 4I). One example from data collected from the cerebellum and the trigeminal ganglion indicated strong negative enrichment of oxidative phosphorylation, indicating a reduced production of transcripts necessary for the assembly of the electron transport chain and mitochondrial function. Conversely, the striatum and the thalamus demonstrated inverse trends in response to both SARS-CoV-2 and IAV infections, showing an increase in oxidative phosphorylation amongst other metabolic ontologies. These trends were also found to be dynamic as the demonstrated an inverse relationship at 31dpi, with cerebellum and trigeminal ganglion showing an increase in oxidative phosphorylation in contrast to striatum and mPFC, where these signatures become negatively enriched (Fig. 4I). These data suggest a dynamic process of metabolic changes that occur throughout the central nervous system in response to viral challenge.

To better assess the functional impact of viral insult or associated metabolic modulation on neural tissue, ontologies relating to synaptic signaling and neural plasticity were further examined. Changes in synaptic signaling showed distinctive responses to SARS-CoV-2 in the thalamus and cerebellum at 3dpi (Fig. 4I). Similarly, when examining genes associated with synaptic plasticity, we observe down-regulation of these processes predominantly in response to SARS-CoV-2 and primarily only during the acute phase of infection, although unique changes in response to IAV could also be visualized.

Lastly, we examined gene ontologies encompassing aspects of the immune response following either SARS-COV-2 or IAV infection. Like the metabolic signatures, we observe similar responses between SARS-CoV-2 and IAV during acute infection. In line with qRT-PCR data from early infection time points (fig. S4F to I), the host response to SARS-CoV-2 or IAV challenge results in IFN-I signatures across all neural tissues examined, with the exception of thalamus (Fig. 4I). Despite clearance of virus in both model systems and a broad resetting of neuronal and systemic inflammatory programs across surveyed tissues, we observed persistent IFN-I and -II signatures selectively in the olfactory bulb following SARS-CoV-2 infection (Fig. 4I).

### A persistent immune response in the olfactory bulb and epithelium was observed in response to SARS-CoV-2 infection in hamsters.

**Fig. 5.**
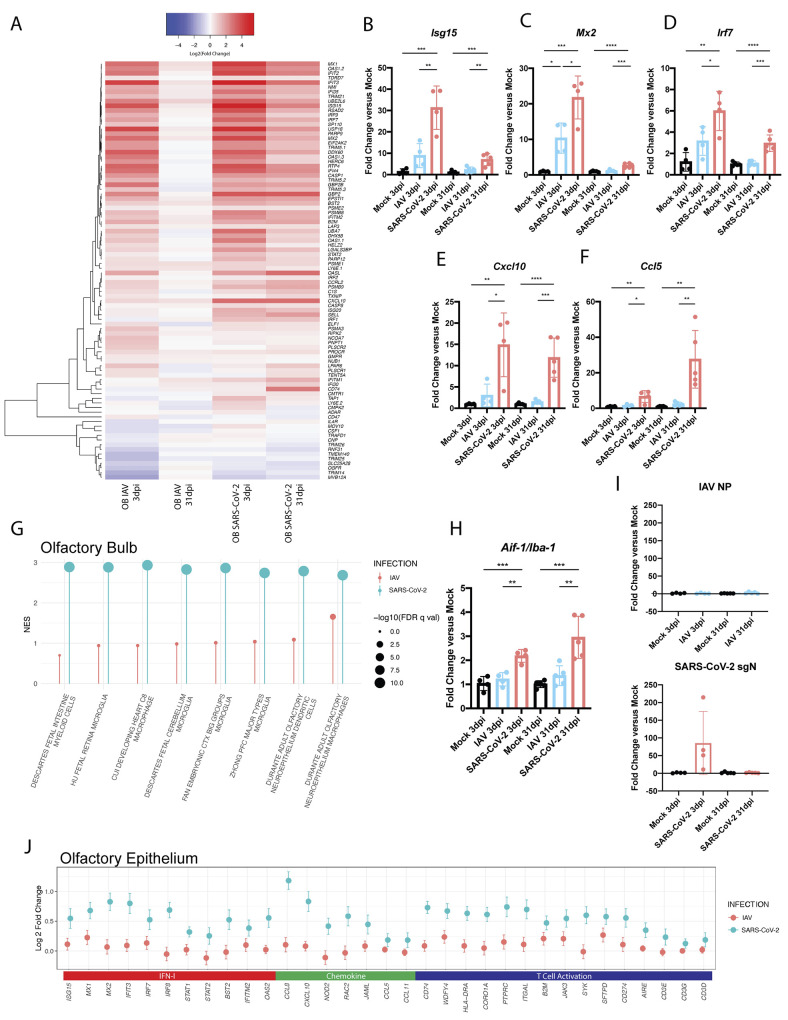
**Persistent inflammation in the olfactory bulb and epithelium is observed in response to SARS-CoV-2 infection in hamsters. (A)** A heat map is shown denoting differential expression analysis conducted on RNA-seq derived from olfactory bulbs of 3dpi and 31dpi IAV- and SARS-CoV-2-infected hamsters compared to mock-infected hamsters. Log2 fold-change of IFN-I response genes are displayed. **(B to F)** Expression of key IFN-I response associated genes (**B**) *Isg15*, (**C**) *Mx2*, and (**D**) *Irf7*; chemokines (**E**) *Cxcl10*, and (**F**) *Ccl5* were quantified by qRT-PCR (n=4 to 5 per treatment and time point group). Error bars display standard deviation, and significance was determined by independent tests on samples from 3dpi and 31dpi for each gene using one-way ANOVA with Tukey’s multiple comparisons test; *p<0.05, **p<0.01, ***p<0.001, ****p<0.0001. **(G)** Differential expression data derived from olfactory bulbs of hamsters 31dpi was analyzed by GSEA analysis to deconvolute specific cellular identity signatures. Top positive enrichments are denoted by lollipop chart based on their NES (magnitude of line) and significance (-log10(FDR q-val)) (dot size). (CTX: Cortex; PFC: Prefrontal Cortex) **(H)** Expression of *Aif-1* (also known as *Iba1*) was quantified by qRT-PCR (n=4 to 5 per treatment and time point group). Error bars display standard deviation, and significance was determined by independent tests on samples from 3dpi and 31dpi for each gene using one-way ANOVA with Tukey’s multiple comparisons test; **p<0.01, ***p<0.001. **(I)** Concentrations of IAV or SARS-CoV-2 RNA in the olfactory bulbs were measured by qRT-PCR with primers for IAV nucleoprotein (IAV NP) or SARS-CoV-2 subgenomic nucleocapsid protein (SARS-CoV-2 sgN), respectively (n=4 per treatment and time point group). Error bars display standard deviation. **(J)** Olfactory epithelium samples from mock-, IAV-, or SARS-CoV-2-infected hamsters were harvested and transcriptionally profiled using RNA-seq. Differential expression analysis was conducted on infected groups compared to mock. Log2 Fold Change for expression of individual genes relevant to ontologies concerning IFN-I, chemokine, and T cell activation are presented in the graph for both IAV and SARS-CoV-2 compared to mock; error bars denote standard error.

Given the unique prolonged nature of the proinflammatory response in the olfactory bulb to SARS-CoV-2, we next examined specific genes driving this transcriptional program (Fig. 5). Comparing genes implicated in the IFN-I signature induced by either IAV or SARS-CoV-2 at 3 and 31dpi highlighted the unique persistence of this transcriptional signature in response to SARS-CoV-2 (Fig. 5A). These data demonstrated prolonged elevation of canonical ISGs such as *Isg15*, *Mx2*, and *Irf7,* which were independently corroborated by qRT-PCR (Fig. 5B to D). To further confirm these findings, we performed immunostaining for MX1 on sections taken from the olfactory bulbs of hamsters, either mock-treated or infected with SARS-CoV-2 or IAV at 3 and 31dpi (fig. S5A). These data supported our transcriptome findings and demonstrated elevated MX1 at both 3 and 31dpi in response to SARS-CoV-2, with immunolabeling remaining in the periphery of the olfactory bulbs (fig. S5A). In addition to ISGs, SARS-CoV-2 infection was also found to induce prolonged chemokine induction as shown by elevated expression of *Cxcl10* and *Ccl5,* amongst others (Fig. 5E and F and fig. S5B).

We next sought to determine the composition of immune cells participating in the prolonged inflammatory response. To this end, we analyzed transcriptomic data from olfactory bulbs at 31dpi to identify enriched gene sets that enable deconvolution to identify the specific cell types present. These analyses showed profound enrichment for microglial and myeloid lineage gene sets, specifically in the SARS-CoV-2-infected hamsters at this time point (Fig. 5G). This analysis was further supported by a directed GSEA which identified an enrichment in markers for microglial activation (fig. S5C). To better assess how central nervous system (CNS)-specific cell types were changing in response to SARS-CoV-2 infection at this time point, gene sets were created for neuronal and glial cell populations using previously described cell-type markers (*56*). These efforts further demonstrated enrichment of microglial-specific transcriptomic signatures in olfactory bulbs from SARS-CoV-2-infected hamsters at 31dpi (fig. S5D). In contrast to immune cells, gene sets identifying neuronal populations demonstrated negative enrichment at this same time point, in agreement with studies focused on the molecular basis of anosmia (*57*).

To further corroborate the transcriptional signatures of the olfactory bulbs in response to either SARS-CoV-2 or IAV, we performed independent qRT-PCR validation and immunohistochemistry on genes for which commercial antibodies for the hamster were available. These efforts illustrated a recruitment of microglial and macrophage populations to the olfactory bulbs, as measured by *Aif-1* transcripts, uniquely in response to SARS-CoV-2 (Fig. 5H). We next aimed to assess myeloid cell activation using histology. Immunostaining for IBA-1, the protein encoded by *Aif-1*, demonstrated increased myeloid positivity around the periphery of the olfactory bulb, implicating a role for both microglia and possibly infiltrating macrophages consistent with what has been reported previously during acute infection (*57*). This finding was most pronounced in olfactory bulbs from SARS-CoV-2-infected hamsters at 3dpi but could still be seen at 31dpi (fig. S5E and F). Microglia and infiltrating macrophages expressing IBA-1 could be visualized clustering around the periphery of the olfactory bulbs in both SARS-CoV-2- and IAV-infected hamsters, showing darker staining for IBA-1 and more enlarged and rounded cell bodies (fig. S5F). Based on transcriptional signatures, SARS-CoV-2 infection resulted in greater activation of IBA-1 stained cells, further supporting the enhanced inflammatory state as compared to IAV (fig. S5C) (*58*).

In addition to implicating microglial and myeloid lineages in the inflammatory phenotype observed in response to SARS-CoV-2, a positive enrichment of T cell signatures was also noted from these samples. To assess the degree of T cell infiltration involved in the hamster response, olfactory bulbs from 31dpi were immunolabeled for CD3 (fig. S5G). Staining was noticeably sparse, with minimal numbers of cells (about 20 to 50 cells/bulb) labeled positive in mock, IAV, and SARS-CoV-2 olfactory bulb cross-sections, suggesting a lack of T cell contribution in this tissue.

To determine if sustained IFN-I or chemokine expression was the product of a chronic infection, we next performed qRT-PCR on the olfactory bulbs (Fig. 5I). These data clearly demonstrate that at 31dpi, neither IAV nor SARS-CoV-2 transcripts could be detected, although SARS-CoV-2 sgN RNA was evident at 3dpi. qRT-PCR data was further validated by RNA in situ hybridization, which confirmed that SARS-CoV-2 spike protein staining was only observed at 3dpi in the glomerular region and was undetectable at 31dpi (fig. S6A). To further assess whether inflammation of the olfactory bulbs was associated with cellular apoptosis in this region, we performed a terminal deoxynucleotidyl transferase dUTP nick end labeling (TUNEL) stain on mock-, SARS-CoV-2-, or IAV-infected hamsters at these times. Quantification of the total number of TUNEL-positive nuclei in the olfactory bulbs revealed no differences between the infection groups at either time point, indicating that neither the acute infection, nor the uniquely prolonged inflammatory patterns in SARS-CoV-2 olfactory bulbs, were associated with local apoptosis, in agreement with the findings of others (fig. S6B) (*57*).

To explore whether this proinflammatory signal was present in additional anatomical regions linked to the olfactory bulb, the olfactory epithelium was harvested from hamsters infected with SARS-CoV-2, IAV, or PBS (mock) at 31dpi, as this tissue has been demonstrated to harbor infectious virus (*21*, *37*). Ontological analyses of RNA-seq data demonstrated that, similar to the olfactory bulbs, the olfactory epithelium of SARS-CoV-2-infected hamsters uniquely showed up-regulated signatures for IFN-I and IFN-II (Fig. 5J and fig. S6C). These signatures were driven by expression of canonical ISGs such as *Isg15, Mx1, Mx2, Ifit3, Irf7, Oas2,* and *Bst2*. However, in contrast to the olfactory bulb, ontological analysis also highlighted several transcriptomic signatures implicating T cell recruitment, activation, differentiation, and immune response in the olfactory epithelium. Chemotactic recruitment signatures were driven by increases in expression of *Ccl7, Cxcl10, Ccl5,* and *Ccl11* as well as other cellular migration factor genes, such as *Jaml* and *Rac2* (Fig. 5J and fig. S6C). T cell activation ontologies, on the other hand, were driven by up-regulated expression of antigen presentation markers, such as *Hla-dra, Wdfy4,* and *B2m,* concurrently with up-regulation of T cell-associated genes such as *Jak3, Coro1A, Cd3e, Cd3g,* and *Cd3d* (Fig. 5J and fig. S6C). In addition to immune signatures, these analyses highlighted a negative enrichment for genes relating to sensory perception of smell and olfaction capabilities which were evident for both SARS-CoV-2- and IAV-infected hamsters (fig. S6C).

To better understand the cellular make-up of this immune response, cell type enrichment analyses were again conducted (fig. S6D). These analyses implicated the presence of unique neuroepithelium lymphocytes and macrophage populations in the olfactory epithelium following SARS-CoV-2 infection. Importantly, as SARS-CoV-2 has demonstrated sex-dependent biases, we also assessed whether evidence for sustained perturbations in the olfactory bulbs or epithelium were present in female hamsters (*59*). To this end, a cohort of all female hamsters were infected with SARS-CoV-2 or IAV and analyzed about two weeks post viral clearance (24dpi, fig. S6E and F). Consistent with our earlier results performed in male hamsters, we found elevated *Isg15* and *Ccl5* transcripts in both tissues, suggesting that the observed phenotype was not sex-dependent.

### Olfactory inflammation was associated with behavioral alteration in hamsters.

**Fig. 6.**
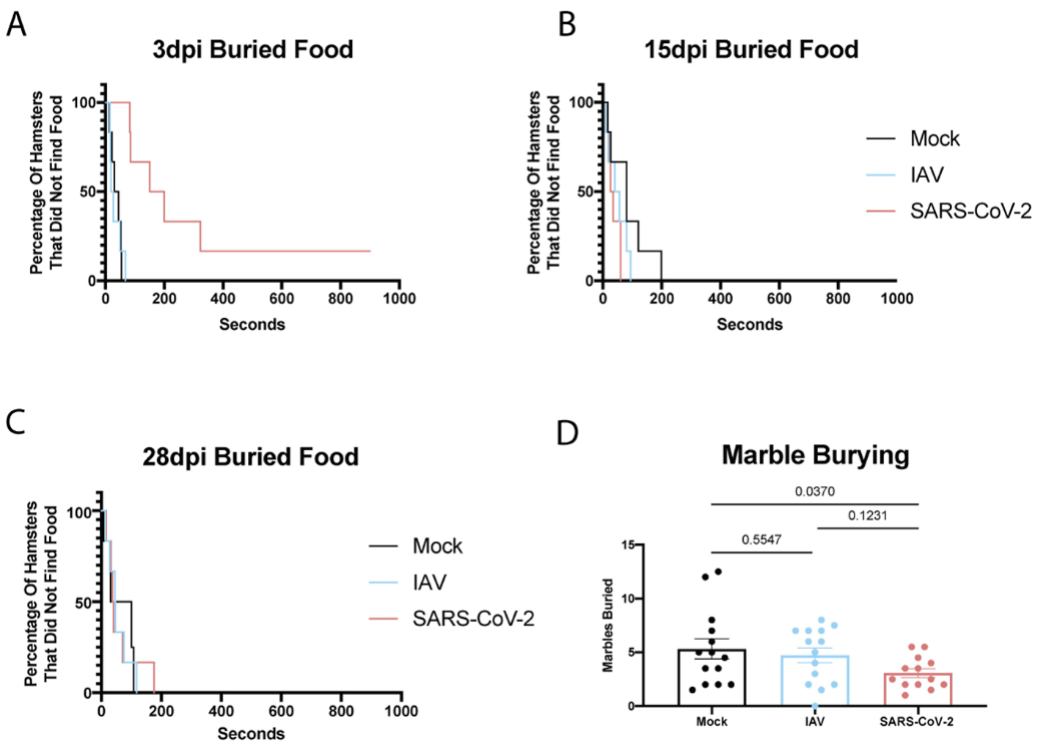
**Olfactory inflammation is associated with behavioral alteration in hamsters. (A to C)** IAV-, SARS-CoV-2, and mock-infected hamsters were assessed for smell at **(A)** 3dpi, **(B)** 15dpi, and **(C)** 28dpi using a buried food finding test (n=6 per treatment group per time point). Kaplan-Meier curves demonstrate time to discovery of food across all time points and infection groups. **(D)** All infection groups were assessed for behavior at 26dpi using the marble burying assay, a test classically utilized to measure repetitive, obsessive-compulsive, and anxiety-like behavior in rodents. The number of marbles that were greater than 60% buried were counted and graphed for each hamster. P-values plotted above the graph were calculated for pairwise comparisons using an ordinary one-way ANOVA with Fisher’s Least Significant Difference test. Outliers were corrected for using iterative Grubb’s test. n=1 sample was removed from the SARS-CoV-2 group (with outlier removed: n=14 Mock-infected, n=14 IAV-infected, and n=13 SARS-CoV-2-infected hamsters). Error bars indicate standard error mean.

Given prior findings that hamsters, similar to humans, can experience anosmia in response to SARS-CoV-2 infection (*34*), and the fact that injury to the olfactory bulb has been linked to development of neurobehavioral disorders such as depression (*60*–*63*), we next assessed the functional consequences of sustained neuronal perturbations, such as prolonged olfactory bulb and epithelium inflammation in SARS-CoV-2-infected hamsters beyond 4 weeks post-infection. To this end, we first looked to elucidate how SARS-CoV-2 infection affected olfaction. Hamsters infected with IAV or SARS-CoV-2 were assessed for smell and compared to a cohort of mock-infected animals. Utilizing a food-finding test at 3dpi, 15dpi, and 28dpi, we confirmed the results reported in de Melo *et al*., showing SARS-CoV-2-infected hamsters took longer to find buried food at 3dpi, but showed no difference at 15 or 28dpi when compared to mock or IAV (*34*) (Fig. 6A to C and fig. S6G to I). In contrast, when this same experiment was performed with readily visible food, all cohorts, at all time points tested, displayed roughly equivalent times (fig. S6J to L). These results occurred alongside prolonged inflammation in both olfactory bulb and epithelium and coincided with transcriptional signatures indicative of diminished expression of olfactory receptor genes for both IAV and SARS-CoV-2, in agreement with the loss of sustentacular (SUS) cells during acute infection being a driver of anosmia (fig. S6C) (*57*).

To determine whether prolonged olfactory bulb inflammation was correlated with altered metrics on assays that assess affective behaviors, mock-, IAV-, and SARS-CoV-2- treated hamsters were subjected to a marble burying assay at 26dpi (Fig. 6D). When hamsters were subjected to this assay, an established metric for assessing rodent repetitive and anxiety-like behaviors (*64*), SARS-CoV-2-infected animals demonstrated a reduction in burying activity compared to the mock, which performed comparably to the IAV-infected group (Fig. 6D). Behavioral tests such as these are believed to reflect a sign of elevated compulsiveness or anxiety-like behaviors (*65*). Given this evidence, this behavior suggests that SARS-CoV-2 induces behavioral changes in hamsters.

### SARS-CoV-2 infection is associated with sustained inflammatory transcriptional programs in human olfactory tissues.

**Fig. 7.**
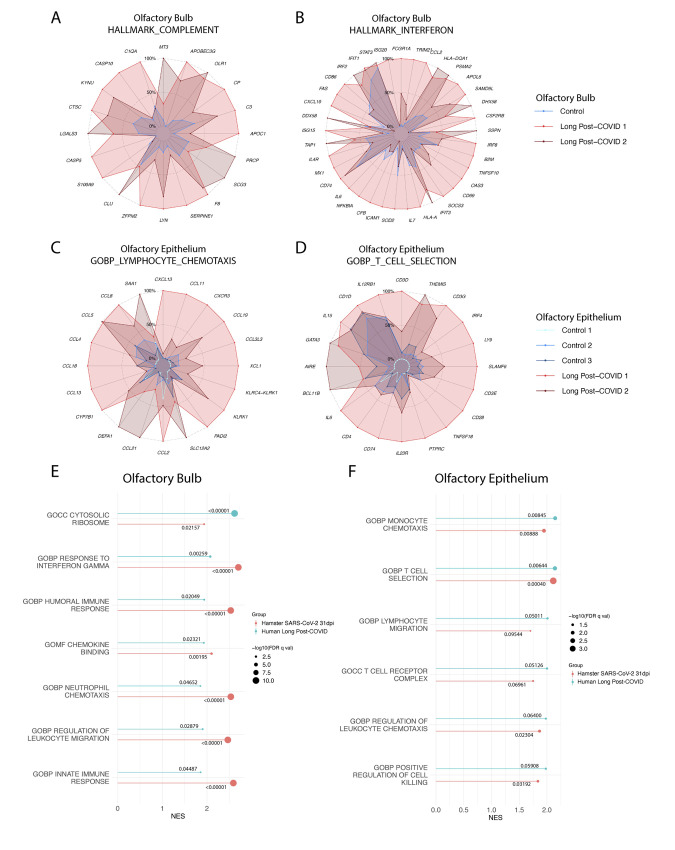
**SARS-CoV-2 infection is associated with sustained inflammatory transcriptional programs in human olfactory bulb and olfactory epithelium. (A and B)** Radar plots derived from olfactory bulb tissues collected at autopsy from healthy control donor (Control) (n=1) as well as donors that had previously recovered from clinically documented COVID-19 (long Post-COVID) (n=2). Donors were screened to only include those where COVID-19 positivity was documented greater than 1 month prior to autopsy. Tissues were RNA-sequenced, and Long Post-COVID tissues were compared to control tissues by differential expression analysis. GSEA using the Hallmark Gene sets was utilized to characterize transcriptomic programs. Transcripts per million reads (TPM) counts for individual genes making up these responses were plotted onto radar plots. Gene expression is normalized to the highest expressing sample for each individual gene, with expression shown as the percentage of TPM value of that sample (which is shown as 100% of its own value). **(A)** Complement and **(B)** interferon responses were measured. **(C and D)** Analyses as described in (A) were used to characterize the transcriptional response of olfactory epithelium tissues harvested from Long-Post COVID (n=2) and control donors (n=3), evaluating **(C)** lymphocyte chemotaxis and **(D)** T cell selection. **(E and F)** GSEA enrichment plots from **(E)** olfactory bulb and **(F)** olfactory epithelium human tissues were plotted by their NES (magnitude of line) and significance (-log10(FDR q-value)) (size of dot). GSEA enrichments of these same gene sets from analogous tissue analysis in hamsters (SARS-CoV-2-infected versus mock-infected olfactory bulb and epithelium tissues at 31dpi) were plotted beside matching human enrichment data. The numerical FDR q-value of each enrichment is denoted above or below the respective NES line for that gene set.

Finally, to ascertain whether our data could be extended to aspects of the human disease, we performed RNA-seq on post-mortem olfactory bulb and olfactory epithelium tissue (Fig. 7). These tissues derived from donors that had recovered from a medically documented history of COVID-19 infection, defined as being PCR negative for more than 1 month prior to death (tables S1 and 2). Tissues from healthy donors without history of COVID-19 infection were also collected as controls.

For olfactory bulb tissues, two COVID-19 recovered (long post-COVID) donors and one uninfected control donor were able to be sequenced. Differential expression and gene enrichment analyses revealed the presence of proinflammatory transcriptional programs in the olfactory bulbs of the recovered donors compared to control tissues (Fig. 7A and B, table S1). In agreement with that observed in hamsters, gene sets for complement (Fig. 7A) and interferon (Fig. 7B) were enriched. Complement gene set enrichment was driven by up-regulation of direct complement cascade genes such as *C3, F8,* and *C1QA* as well as complement regulatory proteins *S100A9, SERPINE1, and CLU* (Fig. 7A). Interferon ontologies were driven by shared up-regulation of ISGs such as *ISG15, OAS3, ISG20, CXCL10, MX1, IFIT3, IFIT1,* and *IRF2*, as well as other immune-related genes such as those involved in antigen presentation (*B2M, HLA-DQA1, CD74*) and cytokine signaling (*IL7, IL6*, *IL4R*) (Fig. 7B). Of note, one long post-COVID donor (Long Post-COVID 1) showed elevated inflammatory gene expression than the other, possibly reflecting their medical history as this individual had COVID-19 within four months of death as opposed to the companion sample (about six months).

For olfactory epithelium tissues, two additional long post-COVID donors and three uninfected control donors were able to be successfully sequenced. Differential expression and gene enrichment analyses showed that, similar to findings in olfactory bulb tissues, long post-COVID donor tissue displayed enriched proinflammatory transcriptional profiles (Fig. 7C and D, table S2). In contrast to interferon-mediated transcriptional programs observed in the OB, the OE displayed a higher enrichment for gene sets detailing chemotactic and T cell-specific activities. These programs were respectively driven by up-regulation of a variety of chemotactic genes (such as *CCL5, CCL8, CCL19, CXCR3*) and T cell-associated genes (such as *CD3D, CD3G, CD3E, GATA3, CD4, LY9*) (Fig. 7C and D). Once again, one long post-COVID donor (long Post-COVID 1) appeared to have a higher abundance of chemotactic and T cell markers than the other long post-COVID sample, likely reflecting the time between COVID-19 and death.

Comparison of the olfactory bulbs and epithelium from 31dpi derived from SARS-CoV-2-infected hamsters and long post-COVID humans demonstrated a correlation between the respective transcriptional programs. In the olfactory bulb, both hamster and human SARS-CoV-2-recovered tissues show enhanced induction of IFN-II, leukocyte chemotaxis, and immune response pathways (Fig. 7E). The two organs further show coordinated metabolic programs, with both hamster and human post-SARS-CoV-2 infection tissues demonstrating up-regulation of ribosomal production (Fig. 7E). Moreover, in the olfactory epithelium, both human and hamster tissues demonstrate enrichment of chemotaxis and T cell functional pathways after SARS-CoV-2 clearance (Fig. 7F). The notable correlation generated when comparing transcriptional responses between hamsters and humans that have recovered from SARS-CoV-2 would suggest that the host response results in prolonged olfactory inflammation capable of impacting other areas of the brain.

## DISCUSSION

Together, these data demonstrate that SARS-CoV-2 and IAV infections produce a wide range of longitudinal systemic impacts that include both shared and unique characteristics. In peripheral tissues such as lung, heart, and kidney, SARS-CoV-2 and IAV seem to induce similar transcriptomic and histological changes both during active infection and following viral clearance - highlighting the importance of benchmarking the host response to SARS-CoV-2 against an independent virus challenge model before accrediting a phenotype as distinct. Of note, despite the strength of modeling the host response to two different viruses, the trajectory of replication of SARS-CoV-2 versus IAV did restrict our comparisons to the initial peaks of both infections (day 3) and the weeks beyond virus clearance (day 14 to 31). The times between these windows involve varying rates of replication and clearance, which made direct comparisons of temporally matched samples difficult to interpret. When focusing on day 3 during the acute infection peak of both infection models, transcriptional profiles were dominated by systemically up-regulated interferon signatures, indicating a robust cell-intrinsic antiviral response across nearly all tissues in response to both viruses. Despite this, histological characterization of tissues outside of the lungs during acute infection showed minimal evidence of cellular infiltration. It is important to note that, although kidney immune infiltration and markers of viral presence have been observed in human patients, these findings have almost exclusively been documented in post-mortem patients that died of severe COVID-19 infection. Our findings here thus likely reflect a milder infection state (*66*). Following resolution of the infection, scarring from SARS-CoV-2 infection were more severe than that observed for IAV. This severity involved a higher degree of kidney tubular atrophy and lambertosis compared to IAV. These differences likely reflect the unique biologies of each virus. SARS-CoV-2 generates substantially more double stranded RNA (dsRNA) during its life cycle as a result of sgRNA production (*13*, *67*). Given the immunogenicity and stability of dsRNA, it seems reasonable to postulate that similar degrees of replication would result in a more robust immune response to SARS-CoV-2 as compared to IAV. Perhaps it is the unique magnitude of the host response to SARS-CoV-2 that induces the observed pathological abnormalities which would result in reduction of functional capacity in affected regions, as supported by previously reported data (*45*, *47*, *68*–*71*). Together with data indicating that SARS-CoV-2 induces a more prolonged inflammatory profile alongside changes in appetite, these data suggest that SARS-CoV-2 induces a more severe systemic acute infection than IAV, which our data here indicates is likely mediated by inflammatory processes shared between the two viruses (*36*, *37*, *72*).

Similar to peripheral tissues, the nervous system showed a mix of shared and unique responses to SARS-CoV-2 and IAV infection. During acute infection, both viruses induced CNS-wide IFN-I responses as well as region-specific transcriptional alterations that in some cases persisted beyond one month following infection. The most prominent of these alterations took place in the striatum, where both IAV and SARS-CoV-2 induced similar changes associated with metabolic and functional shifts. Pre-clinical and clinical literature have correlated this type of activity within striatal subregions with chronic or traumatic stress (*73*, *74*), affective disorders (*75*, *76*), and chronic pain states (*77*). These changes could partially underlie the increased clinical risk of neurological and neuropsychiatric disorder onset associated with IAV (*78*), SARS-CoV-2 (*79*, *80*), and even other viruses and seemingly unrelated clinical conditions (*81*, *82*).

In contrast to the striatum, the thalamus displays a differentially regulated response to SARS-CoV-2 and IAV infection, showing a hypoexcitable versus a hyperexcitable state, respectively. The hypoexcitable state induced by SARS-CoV-2 in the hamster model also show many similarities to clinical reports concerning cognitive deficits and dysexecutive syndrome amongst patients with COVID-19 (*83*, *84*). These data suggest that thalamic dysregulation may contribute to cognitive disruption, potentially in the form of altered intra-thalamic function or functional connectivity with key brain regions that drive emotion, motivation, cognition, sleep, pain, wakefulness, and motor activity. Altered thalamic function and structure has also been previously associated with cognitive deficits in conditions such as multiple sclerosis (*85*), traumatic brain injury (*86*), and Alzheimer’s disease (*87*). Thalamic dysfunction may also underlie neurological conditions that have been observed in patients with long COVID including chronic pain, headache, myalgias, seizures, sleep, and affective disorders (*88*–*94*). Furthermore, transcriptomic changes strongly associated with dendrite development were also seen in this region at both early and late time points after SARS-CoV-2-infection but not in response to IAV-infection. Intriguingly, dysregulation of the key genes driving this enrichment in the SARS-CoV-2 thalamus (*Nrp1, App, Crtc1, Ctnnd2, Camk2a, Kalrn, Bmp7, Ppp1r9b, Mecp2, Cux1, Dlg4, Apoe, Ephb2, Map2k7,* and *Ephb1)* are associated with cognitive impairments and affective disorders such as major depressive disorder when analyzed together using Enrichr’s DisGeNET function (*95*). Thus, regional transcriptional changes in thalamic nuclei may facilitate the development of neuropsychiatric disorders in patients recovering from SARS-CoV-2 infection.

By far, the most unique response to SARS-CoV-2 took place in the olfactory tissue. At 31dpi, the olfactory bulb of IAV-infected hamsters returned to a baseline transcriptional state, whereas SARS-CoV-2-infected hamsters appeared to be in the midst of an ongoing infection characterized by microglial and infiltrating macrophage activation and a robust IFN-I and chemokine response. This phenotype was robust, consistent, and was present in both male and female hamsters. These inflammatory responses were especially surprising given that we were unable to detect presence of viral RNA in either the olfactory bulb or lungs at this time point and, as demonstrated previously, we know that hamsters generate a strong spike protein-specific antibody response as early as 7dpi (*21*, *37*). Immunohistochemistry of MX1 corroborated our transcriptional findings and showed localization of this persistent IFN-I response to the glomerular regions of the olfactory bulbs. Given the olfactory bulbs were positive for SARS-CoV-2 early in infection, these data may suggest the existence of persistent defective viral genomes or remaining debris capable of inducing a host response. Alternatively, SARS-CoV-2 infection of olfactory bulbs may result in loss of a physical barrier, enabling the introduction of microbiome commensals which could contribute to the inflammatory profile. This latter hypothesis is supported by recent evidence that SARS-CoV-2 can induce gross morphological changes in the olfactory epithelium characterized by thinning and sloughing of the tissue that would normally prevent bacterial commensals from interacting with the OB (*96*). The extent to which the olfaction system contributes to the persistent inflammatory processes described within our study remains to be determined. However, when surveying olfactory tissues from human donors that had recovered from documented COVID-19 infection, but had died of other causes, we observed similar transcriptional signatures to those documented in the hamster model, suggesting a shared etiology. Despite these data, it should be noted that although we were unable to detect infectious material at 31dpi, other reports suggest this may be possible under some circumstances and therefore should not be ruled out as another possible contributor to long COVID symptomology (*21*, *34*, *37*).

Chronic inflammation within the olfactory bulbs can impact sensory, emotional, and cognitive processes. In this study, we see that persistent inflammation in olfactory tissues of SARS-CoV-2-infected hamsters is accompanied by a change in behavior as measured by marble burying. As the olfactory bulbs are functionally connected to—and can thus influence activity of—the limbic system, which controls appetitive, sensory, emotional, and cognitive responses, the connection between these findings suggests this to be the possible causation of changes in behavior. Indeed, prior preclinical studies link olfactory bulb damage with depressive phenotypes that can be reversed with antidepressant treatment (*61*–*63*, *97*). These data suggest that chronic nasal and olfactory bulb inflammation may drive neurodegeneration and structural changes consistent with long COVID symptoms (*60*, *98*). This is further supported by recently reported clinical evidence that shows that patients that have recovered from even mild COVID-19 demonstrate loss of grey matter in limbic cortical areas functionally linked to the olfactory system and by additional clinical evidence demonstrating that long COVID presentation is associated with a variety of immune-associated risk factors (*99*, *100*). Taken together, our peripheral organ and central nervous system findings identify transcriptional and histologic signatures caused by SARS-CoV-2 infection that may induce a variety of somatosensory, affective, and cognitive impairments that persist well past the time of original infection. Given the systemic scope of these findings, we hypothesize that they elucidate a molecular basis of much of the heterogeneous symptomology that makes up long COVID.

Our findings highlight the value in the golden hamster model for its ability to phenocopy COVID-19. However, it should be noted that we do not show direct causality between the persistent inflammation in the brain with the changed behaviors of our hamsters. Moreover, the behavioral tests conducted on our hamsters have largely been characterized in mice and rats. These studies also include fewer animals than generally used for behavioral testing which is a consequence of needing to perform these studies within a high containment facility. Lastly, we were only able to obtain olfactory bulb tissues from a few individuals for comparison to the phenotype observed in the hamster model. These comparisons were not only represented by a small sample size, but we also had to compare the modeling of a moderate infection with hamsters with severe disease outcomes in humans. Although not ideal, the ability to improve on these processes is extremely difficult because patients with moderate COVID-19 do not generally succumb to infection, making sampling of heart, kidney, or other tissues of interest not possible. Given these limitations, we cannot state that all observations documented in hamsters accurately reflect the prolonged symptomology following SARS-CoV-2 infection.

## MATERIALS AND METHODS

### Study Design

The objective of this study was to define the systemic transcriptional response to SARS-CoV-2 and benchmark this data against IAV, both during peak viral load and after clearance. Sample size was dictated by available cage space in our high containment facility which we maximized at every opportunity. Hamsters were always divided into three cohorts: mock treatment, IAV infection, and SARS-CoV-2 infection. In general, we used 3 to 5 animals per experimental variable being tested. Regarding data inclusion and exclusion criteria, all samples were included in the analyses unless technical issues were evident such as low quality sequencing data. In these examples, we re-prepped the sequencing library or excluded samples as necessary. All RNA-sequencing experiments thus included between 2 and 5 animals per tested tissue and condition. Numbers of included animals and samples for sequencing in differential expression analyses can be found on NCBI GEO using accession number GSE203001. Any hamster experiments utilizing numbers of samples that fall outside of this range have numbers noted in figure legends. Human cadaver samples were used only to corroborate our findings in hamsters, and we analyzed as many samples as we could obtain. All histology and behavioral experiments were performed and scored blinded. Marble burying behavioral study data was assessed for outliers which were corrected for using Iterative Grubb’s method. The study was approved by the affiliated institutions of all authors listed. Human heart, lung, and kidney sample sequencing data are available through dbGAP (accession #38851 and ID phs002258.v.1.p1). These samples were originally provided by the Weill Cornell Medicine Department of Pathology. The Tissue Procurement Facility operated under Institutional Review Board (IRB) approved protocol and followed guidelines set by Health Insurance Portability and Accountability Act (HIPAA). Experiments using samples from humans were conducted in accordance with local regulations and with the approval of the IRB at the Weill Cornell Medicine. The autopsy samples were considered human tissue research and were collected under IRB protocols 20-04021814 and 19-11021069. All autopsies had consent for research use from next of kin, and these studies were determined as exempt by IRB at Weill Cornell Medicine under those protocol numbers. Brain tissue and nasal epithelium, including the OE and OB were retrieved under a collaborative effort by the Department of Neuropathology and the Department of Otolaryngology at Columbia University Irving Medical Center. The study was approved by the ethics and Institutional Review Board of Columbia University Medical Center (IRB AAAT0689, AAAS7370). All animal experiments were performed according to protocols approved by the Institutional Animal Care and Use Committee (IACUC: PROTO202000113, IPROTO202100000053) and Institutional Biosafety Committee at the Icahn School of Medicine at Mount Sinai (ISMMS) and New York University Langone Health (NYULH, PROTO202100078).

### Viruses and cells

SARS-CoV-2 isolate USA-WA1/2020 (NR-52281) (Biodefense and Emerging Infections Research Resources Repository, BEI Resources) was propagated in Vero-E6 cells in Dulbecco’s Modified Eagle Medium (DMEM) (Gibco) supplemented with 2% fetal bovine serum (FBS) (Millipore Sigma), 1mM 4-(2-hydroxyethyl)-1-piperazineethanesulfonic acid (HEPES) (Lonza Bioscience) and 1% penicillin/streptomycin (Thermo Fisher Scientific). Virus stocks were filtered by centrifugation with Amicon Ultra-15 Centrifugal filter unit (Sigma-Aldrich) and sequenced to ensure maintenance of the furin cleavage site. Infectious viral titers were quantified by plaque assay in Vero-E6 cells (American Type Culture Collection, ATCC) in DMEM supplemented with 2% FBS, 1mM HEPES and 0.7% OXOID agar (Thermo Fisher Scientific). Assays were fixed in 5% paraformaldehyde (PFA) (Thermo Fisher Scientific) and stained with crystal violet (Sigma-Aldrich). All infections were performed with either passage 3 or 4 SARS-CoV-2. Influenza A virus H1N1 isolate A/California/04/2009 (BEI Resources) was propagated in Madin-Darby canine kidney (MDCK) (ATCC) cells in DMEM supplemented with 0.35% bovine serum albumin (BSA) (Thermo Fisher Scientific), filtered and sequenced in a manner comparable to SARS-CoV-2 stocks. All cells used in this study were routinely tested for the presence of mycoplasma using MycoAlert Mycoplasma Detection Kit (Lonza).

### Hamster experiments

Six to 7 week-old male Golden Syrian hamsters (*Mesocricetus auratus*) were obtained from Charles River Laboratories. Hamsters were acclimated to the CDC/USDA-approved Biosafety Level 3 (BSL-3) facility at ISMMS or NYULH for at least 7 days. Hamsters were intranasally infected with PBS (Gibco), 1000 pfu (100μL) of SARS-CoV-2, or 100,000 pfu (100 μL) of H1N1 IAV under ketamine/xylazine anesthesia. Hamsters were euthanized by intraperitoneal injection of pentobarbital and cardiac perfusion with 60 mL PBS. Each tissue was harvested at 1, 3, 4, 7, 14, or 31dpi. Collected tissues were homogenized with PBS or Trizol (Thermo Fisher Scientific) in Lysing Matrix A homogenization tubes (MP Biomedicals) for 40 s at 6 m/s for 2 cycles in a FastPrep 24 5G bead grinder and lysis system (MP Biomedicals) for plaque assay or RNA isolation, respectively. Additional tissues were fixed in 4% PFA for at least 72 hours prior to embedding in paraffin wax blocks for histology. Prior to fixation, lungs were inflated using 1.5 mL of 4% PFA administered by intratracheal catheter (Exel International). An independent female cohort of 6 to 7 week-old female Golden Syrian hamsters was also obtained from Charles River Laboratories and treated in an analogous manner. These hamsters were euthanized at 24dpi, and collected tissues were processed in an identical manner. All animal experiments were performed according to protocols approved by the Institutional Animal Care and Use Committee (IACUC) and Institutional Biosafety Committee at ISMMS and NYULH. Hamsters were randomly assigned to the different treatment groups and all IAV and SARS-CoV-2 infections were performed in the BSL-3 facility.

### qRT-PCR

RNA was isolated from homogenized samples by TRIzol/phenol-chloroform extraction. One μg of total RNA from each tissue was reverse-transcribed into cDNA with oligo dT primers using SuperScript II Reverse transcriptase (Thermo Fisher Scientific). qRT-PCR was performed using primers described in table S3 and KAPA SYBR Fast qPCR Master Mix (KAPA Biosystems) on a LightCycler 480 Instrument II (Roche). Delta-delta-cycle threshold (DDCt) was determined relative to mock-infected control unless otherwise stated (21).

### Hamster RNA-seq

RNA was isolated from homogenized samples by TRIzol/phenol-chloroform extraction. One μg of total RNA from each tissue was enriched for polyadenylated RNA and prepared for next-generation sequencing using the TruSeq Stranded mRNA Library Prep Kit (Illumina) according to manufacturer’s instructions. Prepared libraries were sequenced on an Illumina NextSeq 500 platform. Fastq files were generated with bcl2fastq (Illumina) and aligned to the Syrian golden hamster genome (MesAur 1.0, ensembl) using the RNA-seq Alignment application (Basespace, Illumina). Salmon files were analyzed using the DESeq2 analysis pipeline (*101*). All genes with an adjusted p value (p-adj) of <0.1 were considered DEGs. GSEA studies were performed using the GSEA_4.1.0 Mac App as made available by the Broad Institute and the University of California, San Diego (*102*, *103*). Analyses were conducted on a pre-ranked gene list with ranking statistic calculated from DESeq2 results output as follows: -log10(p-value)*sign(log2FC) (*104*). Unbiased GSEA analyses were conducted against the Hallmark Gene Sets (v7.4), the curated C5 gene ontology and human phenotype ontology gene set (v7.4), and the curated C8 cell type signature gene sets (v7.4) made available by the Molecular Signatures Database (MSigDB). Additional GSEA analyses were conducted on gene sets manually curated from prior publications as described in text. NES and FDR q-values (false discovery rate values adjusted for analysis size) generated by these analyses were visualized using the ggplot2 package. All visualizations of RNA-seq differential expression data were created in R using ggplot2, pheatmap, ComplexHeatmap, and gplots packages. Gene set enrichment plots were adapted from VisualizeRNAseq (https://github.com/GryderArt/VisualizeRNAseq). Radar plots were created using the ggradar2 package (https://github.com/xl0418/ggradar2). Assessment of read coverage of viral genome was conducted using Bowtie2 and IGV_2.8.13 and visualized using ggplot2. Rank-rank scatter plots were created using the RRHO package using the same ranking statistic as was used in GSEA analyses (*105*).

### H&E, Verhoeff Van Gieson, and TUNEL Staining and Quantification

Paraffin-embedded tissue blocks were cut into 5μm sections and mounted on charged glass slides. Sections were deparaffinized by immersion in xylene and rehydrated in decreasing ethanol dilutions. Slides were then stained with hematoxylin (Gill’s formula, Vector Laboratories, Cat #H3401) and eosin Y (Sigma Aldrich, Cat #E4009) according to manufacturer’s instructions. Slides were dehydrated by immersion in increasing concentrations of ethanol, cleared with xylene, and coverslipped (21). Sections were assessed for clinical features by a board-certified pathologist. Images were morphometrically analyzed using QuPath (*106*) and ImageJ (*107*). Randomly sampled tissue regions were generated from digitized lung and kidney histological images. In kidneys, these regions were assessed for average cellular size across each area. In lung sampled areas, lambertosis coverage and airway sizes were manually quantified by treatment-blinded team members. Verhoeff Van Gieson staining was performed on 5μm sections that were cut from paraffin-embedded tissue blocks and embedded on charged glass slides. Slides were stained using ‘Elastic Stain Kit (Verhoeff Van Gieson/EVG Stain)’ kit (Abcam, ab150667) according to manufacturer instructions. Slides were dehydrated by immersion in increasing concentrations of ethanol (Thermo Fisher Scientific), cleared with xylene (Thermo Fisher Scientific), and coverslipped (21). Slides were digitized using Hamamatsu S210 digital slide scanner. All images of slides were captured using NDP.view.2 software (Hamamatsu).

TUNEL staining was performed on 5μm sections that were cut from paraffin-embedded tissue blocks and embedded on charged glass slides. Slides were deparaffinized and processed using the ‘TUNEL Assay Kit –BrdU-Red’ kit (Abcam, ab666110) according to manufacturer instructions. Nuclei were additionally stained with DAPI. Slides were coverslipped and assessed by confocal microscopy. Total number of TUNEL^+^ nuclei per cross-section of tissue were manually tabulated.

### Immunohistochemistry (IHC)

Paraffin-embedded tissue blocks were cut into 5μm sections and mounted on charged glass slides. Sections were deparaffinized by immersion in xylene and rehydrated in decreasing ethanol dilutions. Antigen retrieval was performed for 45 min in IHC-Tek Epitope Retrieval Steamer (Cat #IW-1102) with slides immersed in IHC-Tek Epitope Retrieval Solution (Cat #IW-1100). Tissue was blocked in Tris-Buffered Saline (TBS) (Fisher Scientific) with 10% goat serum (Millipore Sigma) and 1% bovine serum albumin. Primary antibodies (MX-A: Millipore Sigma, MABF938; IBA-1: Wako, 019-19741; CD3: Dako, A0452; MPO: Dako, A0398) was added to slides at the following dilutions: MX-A, 1:100; IBA-1, 1:2500; CD3, 1:1000; MPO: 1:5000. Sections were incubated overnight at 4°C. Slides were washed in TBS with 0.025% Triton X-100 (Thermo Fisher Scientific) prior to immersion in 0.3% hydrogen peroxide (Thermo Fisher Scientific) in TBS for 15 min. Slides were washed once again and horseradish peroxidase (HRP)-conjugated secondary antibody was added at a 1:5000 concentration (Goat anti-mouse: Thermo Fisher Scientific, Cat #A21426; Goat anti-rabbit: Abcam, Ab6721). Slides were washed twice prior to application of 3,3′ diaminobenzidine (DAB) developing reagent (Vector Laboratories, Cat #SK-4105). Slides were dehydrated by immersion in increasing concentrations of ethanol, cleared with xylene, and coverslipped. Slides were digitized using Hamamatsu S210 digital slide scanner. All images of slides were captured using NDP.view.2 software (Hamamatsu).

### Olfaction Assessment

Olfaction was assessed using the buried food finding test as previously described (*34*, *108*). Hamsters were presented with cereal (Coco Krispies, Kellogg’s) five days prior to test; all were consumed within 1 hour. Twenty hours prior to testing, hamsters were food restricted. On the day of testing, hamsters were placed into clean cages with standard bedding and allowed to acclimate for 20 min. After 20 min, hamsters were moved to a holding cage for 2 min while chocolate cereal was buried underneath the bedding in a corner of the testing cage. Hamsters were then moved back to the cage with cereal in it and placed in the opposite corner of the cage as the buried cereal. Hamsters were timed from placement in cage to the time of detection of food (digging in the area of the buried cereal). Hamsters were limited to a 15-min maximum period to find cereal. Once food was found, hamsters were moved back to holding cage for 1 min, and food was placed on top of bedding (visible) in a corner of the test cage during this time. The hamster was then once again placed in the opposite corner of the test cage from the cereal, and time was recorded from placement of hamster in cage to detection of food. All behavioral studies were in compliance with institutional IACUC protocols and took place inside of a biosafety cabinet according to BSL-3 protocols.

### Marble Burying Assay

The marble burying assay was adapted from previously described protocols (*64*). Hamsters were placed into a corner of a cage with clean bedding that had 20 equally-spaced glass marbles placed inside of it. Hamsters were allowed to move freely about the cage for 15 min, at which time they were moved back to their original cage. The number of buried and unburied marbles per cage were tallied by two independent observers and averaged. Partially buried marbles were counted as buried if greater than 60% of the marble was covered with bedding material. All behavioral studies were in compliance with institutional IACUC protocols and took place inside of a biosafety cabinet according to BSL-3 protocols.

### RNA fluorescent in-situ hybridization (RNAscope)

The Fluorescent Multiplex V2 kit (Advanced Cell Diagnostics) was used for RNAscope. Specifically, we used the FFPE protocol as detailed in the RNAscope Multiplex Fluorescent Reagent Kit v2 Assay User Manual. RNAscope probes were as follows: *Rbfox3* (NeuN) for pan-neuronal labeling (Mau-Rbfox3-C1) and the spike gene (*S*) for SARS-CoV-2 labeling (V-nCoV2019-S-C3). Opal dyes (Akoya Biosciences) were used for secondary staining as follows: Opal 690 for C1 and Opal 570 for C3. DAPI was used for nuclear staining. Images were taken on an LSM880 confocal microscope (Zeiss) with identical parameters between mock- and SARS-CoV-2-infected samples.

### Heart, Lung, and Kidney human sample collection

All autopsies are performed with consent of next of kin and permission for retention and research use of tissue. Autopsies were performed in a negative pressure room with protective equipment including N-95 masks; brain and bone were not obtained for safety reasons. All fresh tissues were procured prior to fixation and directly into Trizol for downstream RNA extraction. Tissues were collected from lung, kidney, and the heart as consent permitted. Post-mortem intervals ranged from less than 24 hours to 72 hours (with two exceptions - one at 4 and one at 7 days - but passing RNA quality metrics) with an average of 2.5 days. All deceased patient remains were refrigerated at 4°C prior to autopsy.

### Human Heart, Lung, and Kidney RNA-seq

For RNA library preparation, all samples’ RNA was treated with DNAse 1 (Zymo Research, Catalog # E1010). Post-DNAse digested samples were then put into the NEBNext rRNA depletion v2 (Human/Mouse/Rat), Ultra II Directional RNA (10ng), and Unique Dual Index Primer Pairs were used following the vendor protocols from New England Biolabs. Completed libraries were quantified by Qubit and run on a Bioanalyzer for size determination. Libraries were pooled and sent to the WCM Genomics Core or HudsonAlpha for final quantification by Qubit fluorometer (Thermo Fisher Scientific), TapeStation 2200 (Agilent), and qRT-PCR using the Kapa Biosystems Illumina library quantification kit.

NYGC RNA-seq libraries were prepared using the KAPA Hyper Library Preparation Kit + RiboErase, HMR (Roche) in accordance with manufacturer's recommendations. Briefly, 50 to 200ng of total RNA were used for ribosomal depletion and fragmentation. Depleted RNA underwent first and second strand cDNA synthesis followed by adenylation, and ligation of unique dual indexed adapters. Libraries were amplified using 12 cycles of PCR and cleaned-up by magnetic bead purification. Final libraries were quantified using fluorescent-based assays including PicoGreen (Life Technologies) or Qubit Fluorometer (Invitrogen) and Fragment Analyzer (Advanced Analytics) and sequenced on a NovaSeq 6000 sequencer (v1 chemistry) with 2x150 base pairs, targeting 60M reads per sample.

RNA-seq data was processed through the nf-core/rnaseq pipeline (*109*). This workflow involved quality control of the reads with FastQC (www.bioinformatics.babraham.ac.uk) adapter trimming using Trim Galore! (https://github.com/FelixKrueger/TrimGalore), read alignment with STAR (*110*), gene quantification with Salmon (*111*), duplicate read marking with Picard MarkDuplicates (https://github.com/broadinstitute/picard), and transcript quantification with StringTie (*112*). Other quality control measures included RSeQC, Qualimap, and dupRadar. Alignment was performed using the GRCh38 build native to nf-core and annotation was performed using Gencode Human Release 33 (GRCH38.p13). Differential expression of genes was calculated by DESeq2 using FeatureCounts reads. Differential expression comparisons were done as either COVID high cases versus COVID-negative controls or COVID low cases versus COVID-negative controls for each tissue specifically. SARS-CoV-2 viral load designations were assigned after quantification of normalized reads mapping to the SARS-CoV-2 genome for each donor. Genes were ranked by the following statistic: log10(p-value)*sign(log2FoldChange). Ranked genes were used as input for GSEA on the molecular signatures database (MSigDB).

### Human OB and OE Sequencing

Brain tissue and nasal epithelium, including the OE and OB were retrieved under a collaborative effort by the Department of Neuropathology and the Department of Otolaryngology at Columbia University Irving Medical Center. The study was approved by the ethics and Institutional Review Board of Columbia University Medical Center (IRB AAAT0689, AAAS7370). Nasal tissues, including OE and respiratory epithelium were harvested from the skull base using an en-bloc resection of the anterior skull base including the cribriform plate. OE tissue was isolated from the olfactory cleft, spanning the turbinate and adjacent septal mucosa prior to being preserved in Trizol reagent.

For human OE and OB, RNA was extracted from 10mg of tissue per sample using Direct-zol RNA kit from Zymo Research (Catalog #R2052). After DNAse treatment 50ng to 1μg of total RNA was used to prepare DNA libraries with Truseq RNA Library Prep Kit v2 (Illumina) following manufacturer’s instruction. Libraries were amplified using 14 PCR cycles followed by AMPure XP beads purification. Next, libraries were quantified with Bioanalyser (Agilent Technologies) and Qubit (Invitrogen) and sequenced on NovaSeq 6000 sequencer (Illumina) at Columbia Genome Center.

All resulting fastq files were aligned to the Homo Sapiens genome (GRCh38, RefSeq) using the RNA-seq Alignment application (Basespace, Illumina). Salmon files were analyzed using the DESeq2 analysis pipeline (*111*) genes with a p-adj of <0.1 were considered DEGs. GSEA studies were performed as described above in ‘Hamster RNA Sequencing’.

### Statistical Analysis

Raw, individual-level data are presented in data file S1. All non-RNA-seq statistical analyses, box and bar graphs, and Kaplan-Meyer plots were prepared using GraphPad Prism 9 as described in figure legends. Significance for these analyses was determined utilizing statistical tests including ANOVA with post-hoc analyses. Specific post-hoc analyses and statistical thresholds are described in figure legends. In the marble burying test, all groups were assessed for outliers which were corrected for using Iterative Grubb’s method. RNA-sequencing analysis was conducted using DESeq2. Differentially expressed genes were defined as any genes with a p-adj of less than 0.1. GSEA was performed as described in the methods section; briefly, genes were ranked based on -log10(p-value)/sign(log2[Fold Change]) and assessed for enrichment of various gene sets as described in both figure legends and the text using the MacOS GSEA application (v4.1.0).

## Supplementary Materials

### Other Supplementary Material for this manuscript includes the following:

MDAR Reproducibility Checklist
