# Supplementary material for: SARS-CoV-2 infection in hamsters and humans results in lasting and unique systemic perturbations post recovery
Source: Sci Transl Med. 2022 Jun 7:eabq3059. doi: 10.1126/scitranslmed.abq3059 (PMC9210449; doi:10.1126/scitranslmed.abq3059)
Supplement: Supplementary file 1 — Figs. S1 to S6 Table S1 to S3 [file scitranslmed.abq3059_sm.pdf]

Science Translational Medicine
AAAS

Supplementary Materials for

SARS-CoV-2 infection in hamsters and humans results in lasting and unique systemic perturbations post recovery

Justin J. Frere *et al.*

Corresponding authors: Venetia Zachariou, venetia.zachariou@mssm.edu;
Benjamin R. tenOever, Benjamin.tenOever@nyulangone.org

DOI: 10.1126/scitranslmed.abq3059

The PDF file includes:

Figs. S1 to S6
Tables S1 to S3

Other Supplementary Material for this manuscript includes the following:

MDAR Reproducibility Checklist
Data file S1

SARS-CoV-2 infection in hamsters and humans results in lasting and unique systemic perturbations post recovery

Justin J. Frere[1,2], Randal A. Serafini[3], Kerri D. Pryce[3], Marianna Zazhytska[4], Kohei Oishi[2], Ilona Golynker[2], Maryline Panis[2], Jeffrey Zimering[3,5], Shu Horiuchi[2], Daisy A. Hoagland[1], Rasmus Møller[2], Anne Ruiz[3], Albana Kodra[4], Jonathan B. Overdevest[6], Peter D. Canoll[7], Alain C. Borczuk[8], Vasuretha Chandar[9], Yaron Bram[9], Robert Schwartz[9,10], Stavros Lomvardas[4], Venetia Zachariou[3*], Benjamin R. tenOever[2*]

[1]Department of Microbiology, Icahn School of Medicine at Mount Sinai, New York, NY 10029
[2]Department of Microbiology, New York University, Grossman School of Medicine, New York, NY 10016
[3]Department of Neuroscience, Icahn School of Medicine at Mount Sinai, New York, NY 10029
[4]Mortimer B. Zuckerman Mind, Brain and Behavior Institute, Columbia University, New York, NY 10027
[5]Department of Neurosurgery, Icahn School of Medicine at Mount Sinai, New York, NY 10029
[6]Department of Otolaryngology- Head and Neck Surgery, Columbia University Irving Medical Center, Vagelos College of Physicians and Surgeons, Columbia University, New York, NY 10032
[7]Department of Pathology and Cell Biology, Columbia University Irving Medical Center, Vagelos College of Physicians and Surgeons, Columbia University, New York, NY 10032
[8]Department of Pathology and Laboratory Medicine, Weill Cornell Medicine, New York, NY 10021
[9]Department of Physiology, Biophysics, and Systems Biology, Weill Cornell Medicine, New York, NY 10021
[10]Division of Gastroenterology and Hepatology, Department of Medicine, Weill Cornell Medicine, New York, NY 10021

*To whom correspondence should be addressed: venetia.zachariou@mssm.edu, and Benjamin.tenOever@nyulangone.org

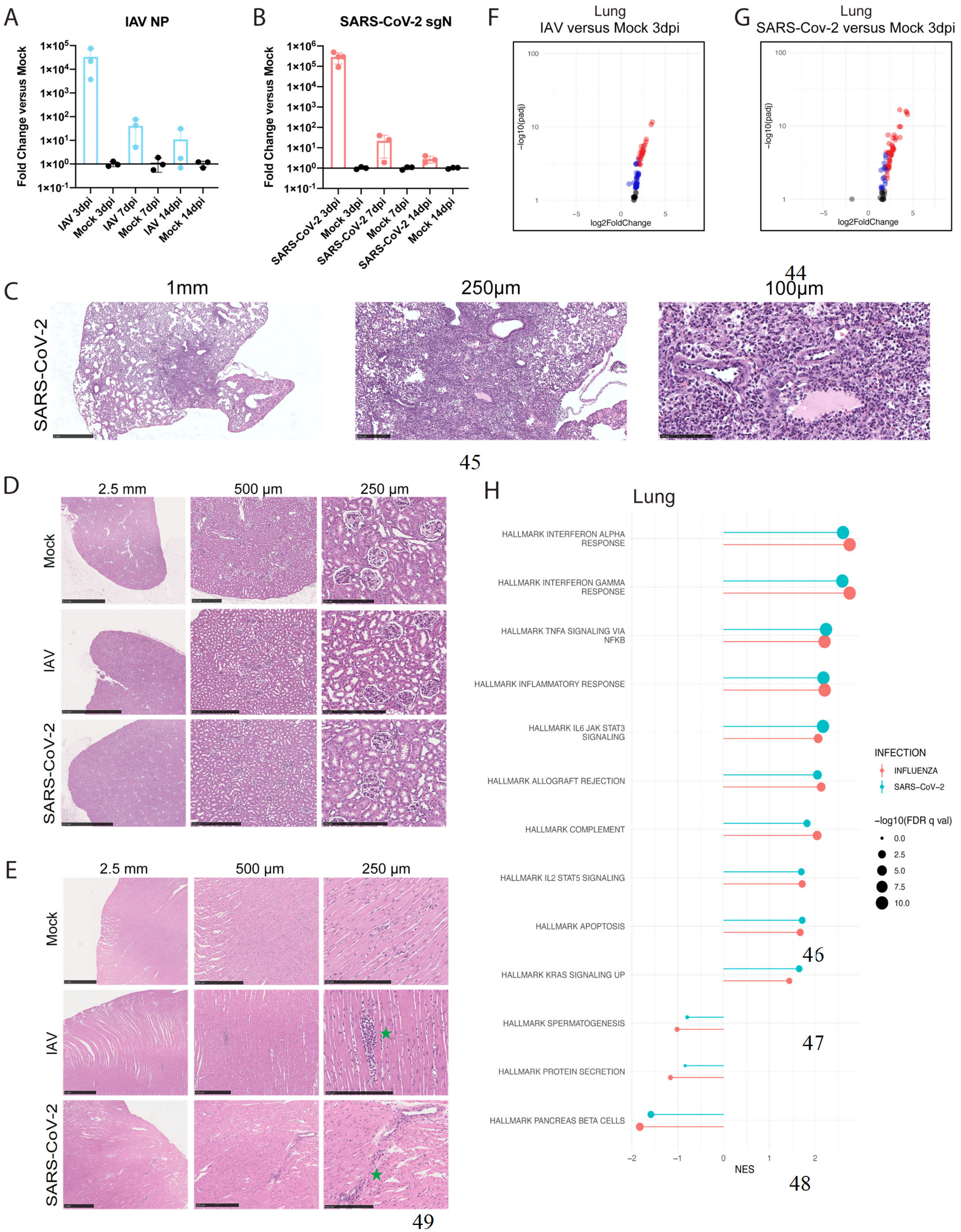

A
IAV NP
Fold Change versus Mock
IAV 3dpi
Mock 3dpi
IAV 7dpi
Mock 7dpi
IAV 14dpi
Mock 14dpi
B
SARS-CoV-2 sgN
Fold Change versus Mock
SARS-CoV-2 3dpi
Mock 3dpi
SARS-CoV-2 7dpi
Mock 7dpi
SARS-CoV-2 14dpi
Mock 14dpi
F
Lung
IAV versus Mock 3dpi
−log10(padj)
log2FoldChange
G
Lung
SARS-Cov-2 versus Mock 3dpi
−log10(padj)
log2FoldChange
C
1mm
250μm
100μm
SARS-CoV-2
D
2.5 mm
500 μm
250 μm
Mock
IAV
SARS-CoV-2
E
2.5 mm
500 μm
250 μm
Mock
IAV
SARS-CoV-2
H
Lung
HALLMARK INTERFERON ALPHA RESPONSE
HALLMARK INTERFERON GAMMA RESPONSE
HALLMARK TNFA SIGNALING VIA NFKB
HALLMARK INFLAMMATORY RESPONSE
HALLMARK IL6 JAK STAT3 SIGNALING
HALLMARK ALLOGRAFT REJECTION
HALLMARK COMPLEMENT
HALLMARK IL2 STAT5 SIGNALING
HALLMARK APOPTOSIS
HALLMARK KRAS SIGNALING UP
HALLMARK SPERMATOGENESIS
HALLMARK PROTEIN SECRETION
HALLMARK PANCREAS BETA CELLS
NES
INFECTION
INFLUENZA
SARS−CoV−2
−log10(FDR q val)
0.0
2.5
5.0
7.5
10.0

Supplemental Legends

fig. S1. SARS-CoV-2 and influenza A virus (IAV) infections in hamsters induce transcriptional and histological changes that mirror human infection pathology.

(A and B) Lungs of an independent longitudinal cohort of hamsters infected with (**A**) IAV (n=3 per time point) or (**B**) SARS-CoV-2 (n=3-4 per time point) were measured for viral load compared to mock treated hamsters (n=3 per time point) by quantitative reverse-transcription-based PCR (qRT-PCR) with primers for IAV nucleoprotein (IAV NP) or SARS-CoV-2 subgenomic nucleocapsid protein (SARS-CoV-2 sgN), respectively.

(C) H&E staining was conducted on lungs of SARS-CoV-2-infected hamsters at 3dpi. Scale bar sizes are denoted above each image.

(D and E) H&E staining was conducted on (**D**) kidneys and (**E**) hearts of SARS-CoV-2-, IAV-, and mock-infected hamsters at 3dpi. Histological analysis of hearts confirmed by board-certified pathologist revealed leukocytic infiltration (green stars).

(F and G) Volcano plots depict differential expression analysis conducted on RNA-seq data derived from lungs of (**F**) IAV- or (**G**) SARS-CoV-2-infected hamsters compared to mock-infected hamsters at 3dpi using DESeq2; differentially expressed genes with a p-adjusted value of less than 0.1 are plotted (black: p-adj > 0.05, log2 fold-change < 2; blue: p-adj < 0.05, log2 fold-change < 2; green: p-adj > 0.05, log2 fold-change > 2; red: p-adj < 0.05, log2 fold-change > 2).

(H) Lollipop charts denoting differential expression data analyzed by Gene Set Enrichment Analysis (GSEA) using the Hallmark gene sets. Charts display normalized enrichment score (NES) and a dot size scaled relative to -log10(FDR q-val) of the enrichment for top ten most positively enriched gene sets and top three most negatively enriched gene sets in this analysis.

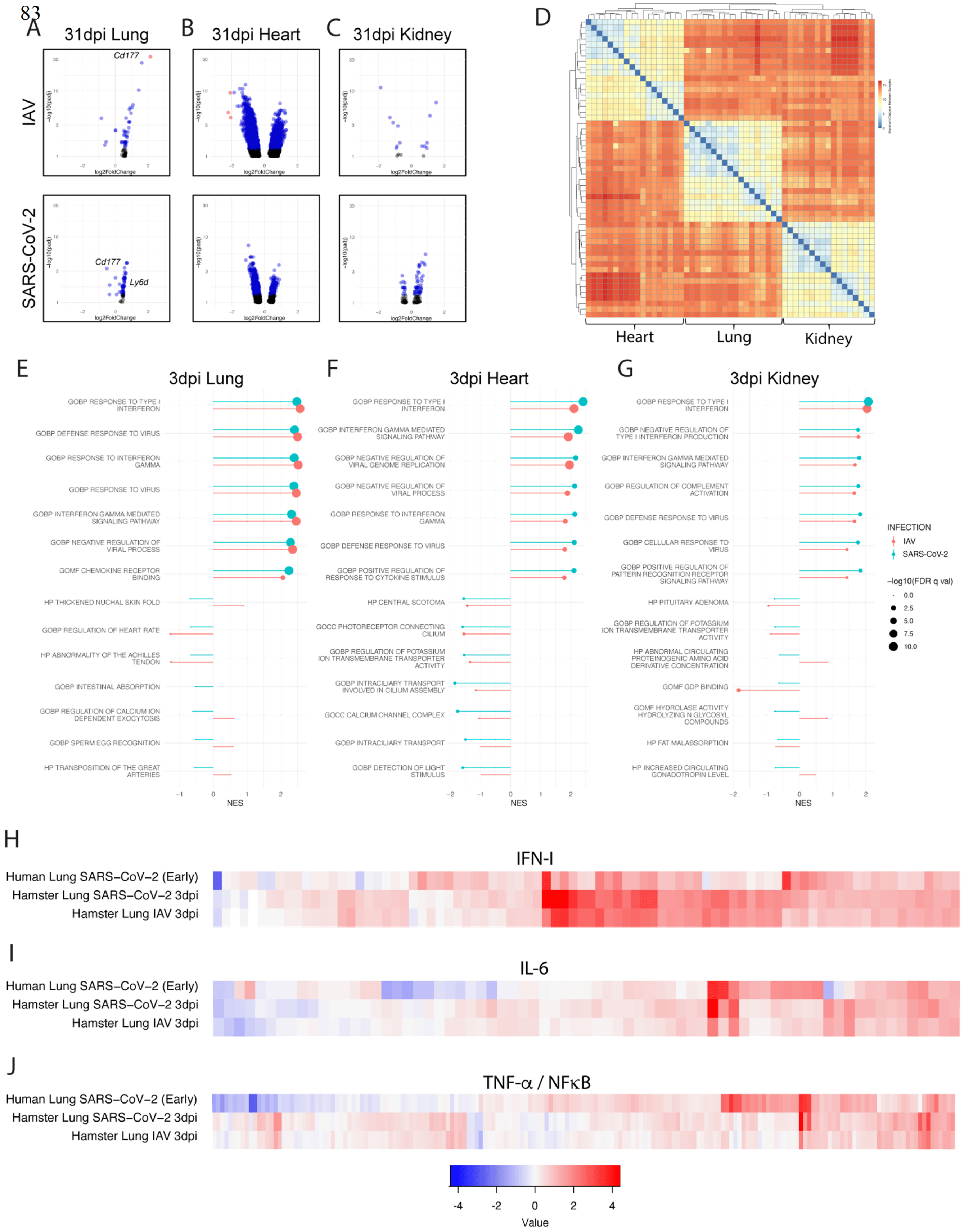
A
31dpi Lung
B
31dpi Heart
C
31dpi Kidney
IAV
SARS-CoV-2
Cd177
Ly6d
log2FoldChange
-log10(padj)
D
Heart
Lung
Kidney
E
3dpi Lung
GOBP RESPONSE TO TYPE I INTERFERON
GOBP DEFENSE RESPONSE TO VIRUS
GOBP RESPONSE TO INTERFERON GAMMA
GOBP RESPONSE TO VIRUS
GOBP INTERFERON GAMMA MEDIATED SIGNALING PATHWAY
GOBP NEGATIVE REGULATION OF VIRAL PROCESS
GOMF CHEMOKINE RECEPTOR BINDING
HP THICKENED NUCHAL SKIN FOLD
GOBP REGULATION OF HEART RATE
HP ABNORMALITY OF THE ACHILLES TENDON
GOBP INTESTINAL ABSORPTION
GOBP REGULATION OF CALCIUM ION DEPENDENT EXOCYTOSIS
GOBP SPERM EGG RECOGNITION
HP TRANSPOSITION OF THE GREAT ARTERIES
NES
F
3dpi Heart
GOBP RESPONSE TO TYPE I INTERFERON
GOBP INTERFERON GAMMA MEDIATED SIGNALING PATHWAY
GOBP NEGATIVE REGULATION OF VIRAL GENOME REPLICATION
GOBP NEGATIVE REGULATION OF VIRAL PROCESS
GOBP RESPONSE TO INTERFERON GAMMA
GOBP DEFENSE RESPONSE TO VIRUS
GOBP POSITIVE REGULATION OF RESPONSE TO CYTOKINE STIMULUS
HP CENTRAL SCOTOMA
GOCC PHOTORECEPTOR CONNECTING CILIUM
GOBP REGULATION OF POTASSIUM ION TRANSMEMBRANE TRANSPORTER ACTIVITY
GOBP INTRACILIARY TRANSPORT INVOLVED IN CILIUM ASSEMBLY
GOCC CALCIUM CHANNEL COMPLEX
GOBP INTRACILIARY TRANSPORT
GOBP DETECTION OF LIGHT STIMULUS
NES
G
3dpi Kidney
GOBP RESPONSE TO TYPE I INTERFERON
GOBP NEGATIVE REGULATION OF TYPE I INTERFERON PRODUCTION
GOBP INTERFERON GAMMA MEDIATED SIGNALING PATHWAY
GOBP REGULATION OF COMPLEMENT ACTIVATION
GOBP DEFENSE RESPONSE TO VIRUS
GOBP CELLULAR RESPONSE TO VIRUS
GOBP POSITIVE REGULATION OF PATTERN RECOGNITION RECEPTOR SIGNALING PATHWAY
HP PITUITARY ADENOMA
GOBP REGULATION OF POTASSIUM ION TRANSMEMBRANE TRANSPORTER ACTIVITY
HP ABNORMAL CIRCULATING PROTEINOGENIC AMINO ACID DERIVATIVE CONCENTRATION
GOMF GDP BINDING
GOMF HYDROLASE ACTIVITY HYDROLYZING N GLYCOSYL COMPOUNDS
HP FAT MALABSORPTION
HP INCREASED CIRCULATING GONADOTROPIN LEVEL
NES
INFECTION
IAV
SARS-CoV-2
-log10(FDR q val)
0.0
2.5
5.0
7.5
10.0
H
IFN-I
Human Lung SARS−CoV−2 (Early)
Hamster Lung SARS−CoV−2 3dpi
Hamster Lung IAV 3dpi
I
IL-6
Human Lung SARS−CoV−2 (Early)
Hamster Lung SARS−CoV−2 3dpi
Hamster Lung IAV 3dpi
J
TNF-α / NFκB
Human Lung SARS−CoV−2 (Early)
Hamster Lung SARS−CoV−2 3dpi
Hamster Lung IAV 3dpi
-4
-2
0
2
4
Value

fig. S2. SARS-CoV-2 and IAV induce lasting transcriptional signatures in peripheral organs that are detectable at 31dpi.

(A to C) Volcano plot depict RNA-seq data conducted on **(A)** lungs, **(B)** hearts, and **(C)** kidneys tissue of IAV-, SARS-CoV-2-, and mock-treated hamsters at 31dpi. Differential expression analysis was computed using DESeq2; differentially expressed genes with a p-adjusted value of less than 0.1 are plotted (black: p-adj > 0.05, log2 fold-change < 2; blue: p-adj < 0.05, log2 fold-change < 2; green: p-adj > 0.05, log2 fold-change > 2; red: p-adj < 0.05, log2 fold-change > 2).

(D) RNA-seq data for all heart, lung, and kidney samples were hierarchically clustered by maximal distance between read data for each sample.

(E to G) GSEA analysis using the MSigDB C5 curated gene ontology set was conducted on 3dpi IAV versus Mock and 3dpi SARS-CoV-2 versus Mock differential expression data for **(E)** lungs, **(F)** heart, and **(G)** kidneys. Top significant ontological enrichments in SARS-CoV-2-associated analyses are plotted by their NES (line magnitude) and significance (-log10(FDR q-value)) (dot size). GSEA enrichment for these same gene sets in IAV versus Mock differential expression data for the same tissue is plotted side-by-side for comparison. GOBP: Gene Ontology Biological Process; GOMF: Gene Ontology Molecular Function; HP: Human Phenotype; GOCC: Gene Ontology Cellular Component.

(H to J) GSEA using the Hallmark gene sets was conducted on RNA-seq differential expression data comparing human early-infection SARS-CoV-2 lung tissue from post-mortem donors to control human tissues for enriched ontologies listed. Heat maps show log2 Fold Change of individual genes comprising the respective gene sets for both hamster and human infection groups compared to control tissues. Gene sets analyzed included (**H**) IFN-I responses, (**I**) interleukin (IL)-6, and (**J**) tumor necrosis factor (TNF)-α and nuclear factor κB (NFκB) signaling.

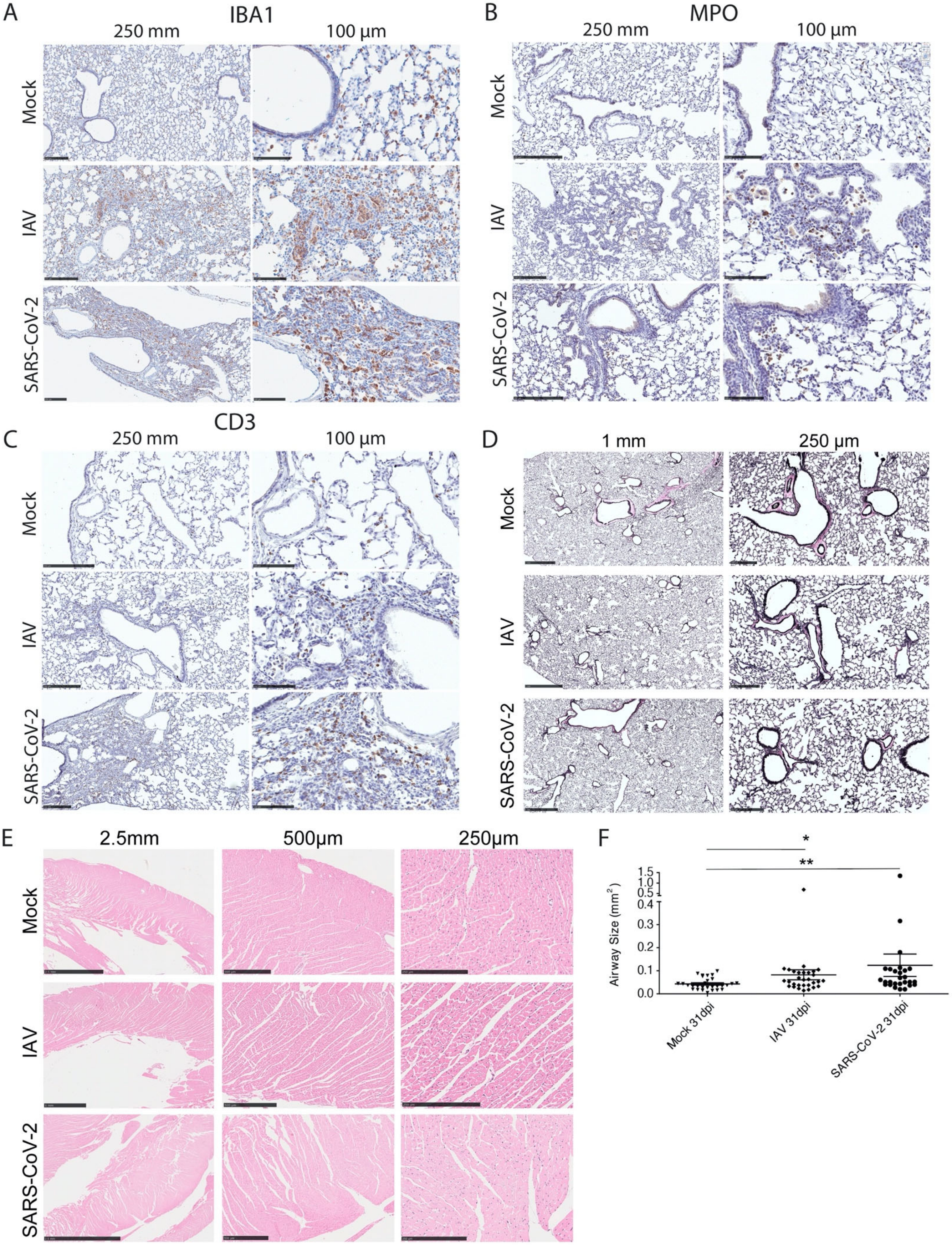

A
IBA1
250 mm
100 µm
Mock
IAV
SARS-CoV-2
B
MPO
250 mm
100 µm
Mock
IAV
SARS-CoV-2
C
CD3
250 mm
100 µm
Mock
IAV
SARS-CoV-2
D
1 mm
250 µm
Mock
IAV
SARS-CoV-2
E
2.5mm
500µm
250µm
Mock
IAV
SARS-CoV-2
F
*
**
Airway Size (mm²)
1.5
1.0
0.5
0.4
0.3
0.2
0.1
0.0
Mock 31dpi
IAV 31dpi
SARS-CoV-2 31dpi

fig. S3. Histological analyses reveal differences between SARS-CoV-2 and IAV-infected hamsters at 31dpi.

(A to C) Immunohistochemical labeling for **(A)** IBA1, **(B)** MPO, and **(C)** CD3 were used to label macrophage, neutrophil, and T cell populations, respectively, in the lungs of mock-, IAV-, and SARS-CoV-2-infected hamsters at 31dpi. Size of inset scale bars matches length described in column headers.

(D) Verhoeff Van Gieson staining was performed on sections derived from the lungs of 31dpi SARS-CoV-2-, IAV-, and mock-infected hamsters.

(E) H&E staining was conducted on hearts of SARS-CoV-2-, IAV-, and mock-infected hamsters at 31dpi.

(F) Airway sizes in cross-sections of lungs from 31dpi SARS-CoV-2-, IAV-, and mock-infected hamsters were quantified by morphometric image analysis (29 Mock, 30 IAV, and 27 SARS-CoV-2 randomly subsampled images of 31dpi lung tissues were analyzed from lung slides generated from 5 hamsters per treatment condition). Error bars display standard error mean, and significance was quantified by one-way ANOVA with Dunn's Multiple Comparison Test; $^{*}p<0.05$, $^{**}p<0.01$.

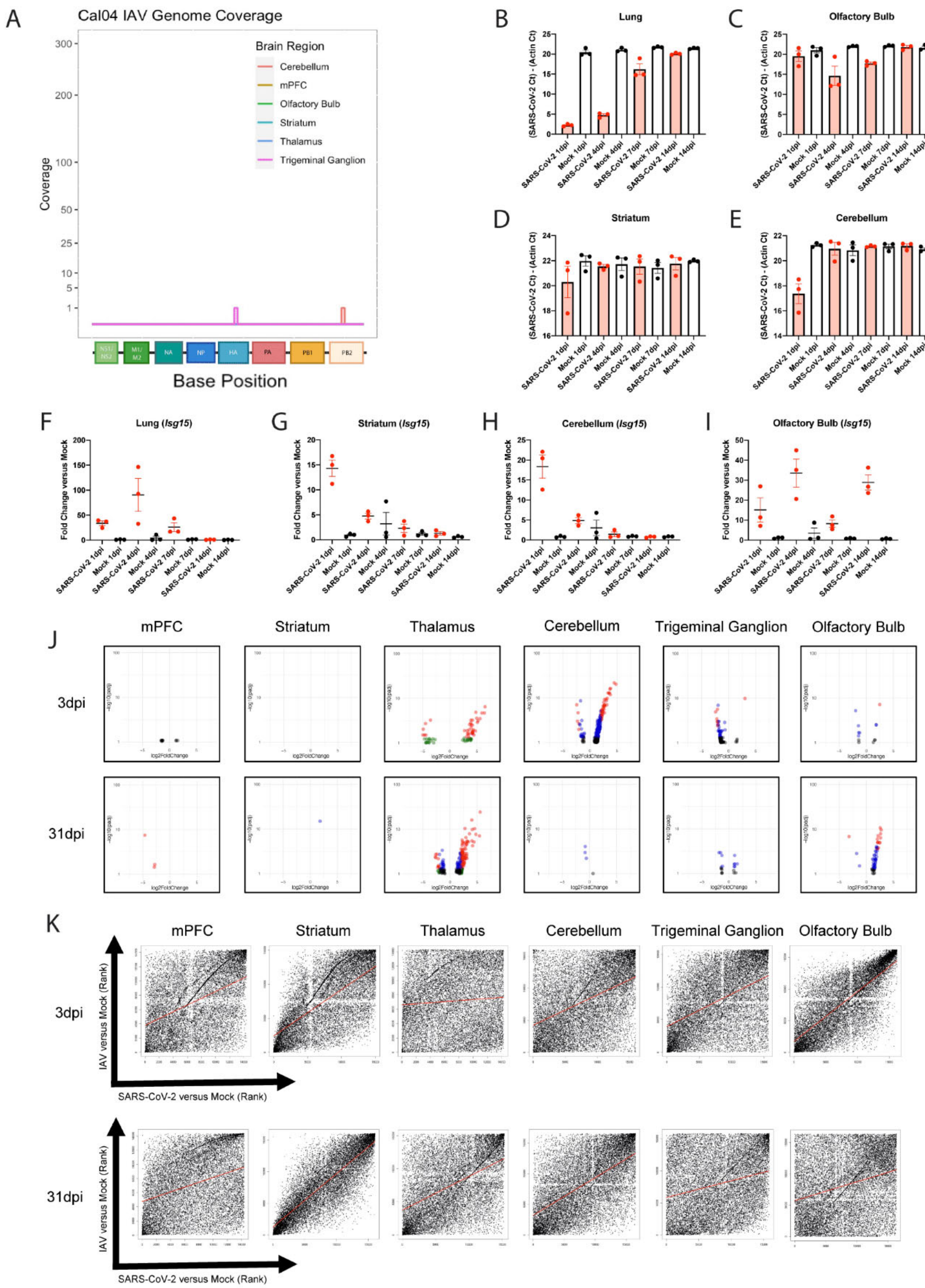

fig. S4. SARS-CoV-2 and IAV induce both unique and shared transcriptional responses in the nervous system.

(A) Olfactory bulbs, medial prefrontal cortex (mPFC), striatum, thalamus, cerebellum, and trigeminal ganglion were bilaterally sampled for RNA-seq analysis from 3dpi IAV-

infected hamsters. Sequencing reads were aligned to the IAV A/California/04/2009 genome, and coverage of raw reads over the length of the genome were displayed as a histogram for each brain region from the hamster with the highest number of reads.

(B) Lungs, **(C)** olfactory bulbs, **(D)** striatum tissues, and **(E)** cerebellum tissues were sampled from a longitudinal SARS-CoV-2- (n=3 per tissue per time point) or mock-infected (n=3 per tissue per time point) hamster cohort at 1, 4, 7, and 14dpi and assessed for SARS-CoV-2 subgenomic nucleocapsid (sgN) protein transcripts by qRT-PCR. Values shown have Ct values for actin (*Actb*), a housekeeping control gene, subtracted from Ct values for sgN for normalization. Error bars denote standard error of the mean.

(F) Lungs, **(G)** striatum tissues, **(H)** cerebellum tissues and **(I)** olfactory bulbs were also assessed for expression of canonical IFN-I gene *Isg15* by qRT-PCR. Values shown display fold change normalized to mock controls. Error bars denote standard error mean.

(J) Differential expression analysis was conducted for SARS-CoV-2-infected hamsters compared directly to IAV-infected hamsters at 3dpi and 31dpi using DESeq2 across the sampled brain regions; differentially expressed genes with a p adjusted value of less than 0.1 are plotted (black: p-adj > 0.05, log2 fold-change < 2; blue: p-adj < 0.05, log2 fold-change < 2; green: p-adj > 0.05, log2 fold-change > 2; red: p-adj < 0.05, log2 fold-change > 2).

(K) Rank-rank scatter plots were generated for each brain region at 3dpi and 31dpi to compare coordination of gene expression from SARS-CoV-2- and IAV-infected hamsters when compared to mock-infected hamsters. Red lines indicate regression fit lines.

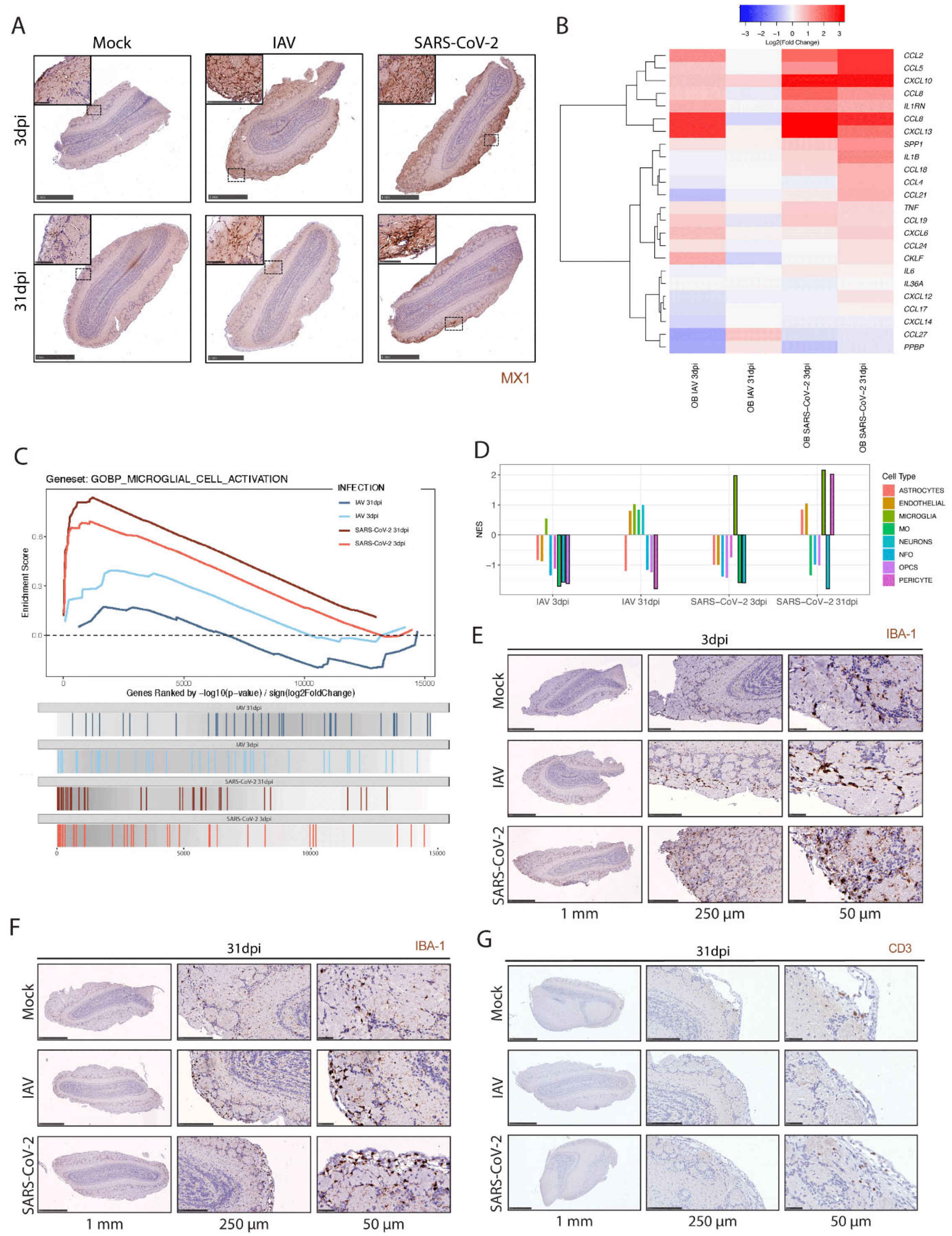

Fig. S5. SARS-CoV-2 induces a uniquely prolonged chemokine signature detectable at 31dpi in the olfactory bulb (OB).

(A) Formalin-fixed paraffin-embedded (FFPE) olfactory bulbs from mock-, IAV-, or SARS-CoV-2-infected hamsters at 3dpi and 31dpi were immuno-labeled for MX1 protein. Zoomed inset displays glomerular area of maximum positivity within each sample. Scale bars indicate 1mm in the main image and 100μm in the insert.

(B) Differential expression analysis was conducted on RNA-seq data of olfactory bulbs of 3dpi and 31dpi IAV- and SARS-CoV-2-infected hamsters compared to mock-infected hamsters. Log2 fold-change of curated chemokine genes from this analysis are displayed in the heatmap.

(C) GSEA of the GOBP_MICROGLIAL_CELL_ACTIVATION ontology was conducted on RNA-seq differential expression data from olfactory bulbs of 3dpi and 31dpi IAV- and SARS-CoV-2-infected hamsters and displayed as a GSEA enrichment plot.

(D) Differential expression data comparing olfactory bulbs of 3 and 31dpi infected hamster cohorts to mock-infected was analyzed for enrichment of cell types using GSEA. Gene sets surveyed in this analysis were created using characterized cell type-specific markers characterized in (*56*). NES values are plotted. Enrichments achieving significance (FDR q-value < 0.05) are outlined in black and were moved to right most side of the sample cluster for easier identification. MO, Myelinating Oligodendrocytes; NFO, Newly Formed Oligodendrocytes; OPCS, Oligodendrocyte Precursor Cells.

(E and F) FFPE olfactory bulbs from mock-, IAV-, or SARS-CoV-2-infected hamsters at **(E)** 3dpi and **(F)** 31dpi were immuno-labeled for IBA-1 protein. Inset scale bar is representative of the length displayed at the bottom of the given column.

(G) FFPE olfactory bulbs from mock-, IAV-, or SARS-CoV-2-infected hamsters at 31dpi were immuno-labeled for CD3 protein. Inset scale bar is representative of the length displayed at the bottom of the given column.

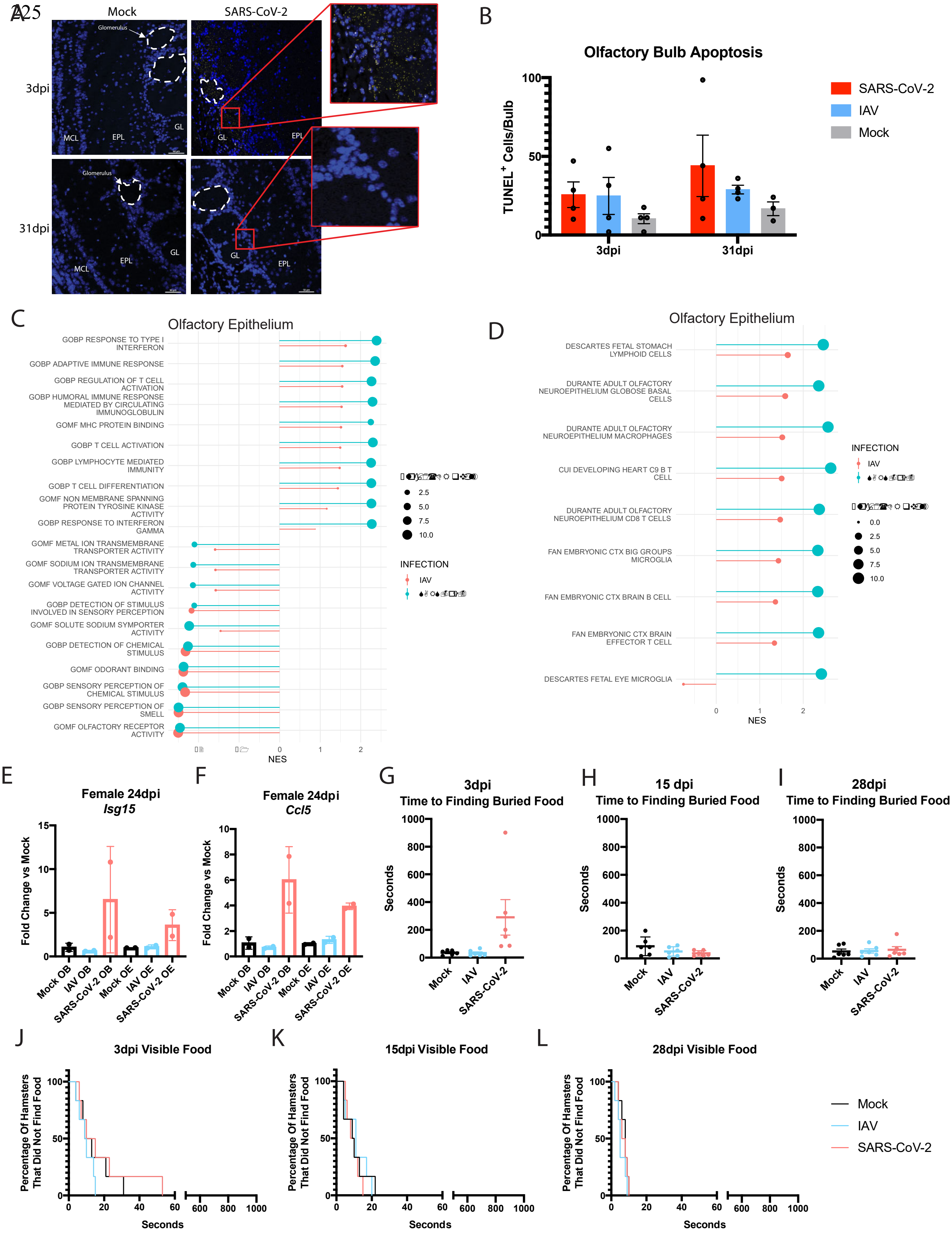
A25
Mock
SARS-CoV-2
3dpi
31dpi
Glomerulus
MCL
EPL
GL
B
Olfactory Bulb Apoptosis
TUNEL + Cells/Bulb
SARS-CoV-2
IAV
Mock
3dpi
31dpi
C
Olfactory Epithelium
GOBP RESPONSE TO TYPE I INTERFERON
GOBP ADAPTIVE IMMUNE RESPONSE
GOBP REGULATION OF T CELL ACTIVATION
GOBP HUMORAL IMMUNE RESPONSE MEDIATED BY CIRCULATING IMMUNOGLOBULIN
GOMF MHC PROTEIN BINDING
GOBP T CELL ACTIVATION
GOBP LYMPHOCYTE MEDIATED IMMUNITY
GOBP T CELL DIFFERENTIATION
GOMF NON MEMBRANE SPANNING PROTEIN TYROSINE KINASE ACTIVITY
GOBP RESPONSE TO INTERFERON GAMMA
GOMF METAL ION TRANSMEMBRANE TRANSPORTER ACTIVITY
GOMF SODIUM ION TRANSMEMBRANE TRANSPORTER ACTIVITY
GOMF VOLTAGE GATED ION CHANNEL ACTIVITY
GOBP DETECTION OF STIMULUS INVOLVED IN SENSORY PERCEPTION
GOMF SOLUTE SODIUM SYMPORTER ACTIVITY
GOBP DETECTION OF CHEMICAL STIMULUS
GOMF ODORANT BINDING
GOBP SENSORY PERCEPTION OF CHEMICAL STIMULUS
GOBP SENSORY PERCEPTION OF SMELL
GOMF OLFACTORY RECEPTOR ACTIVITY
NES
INFECTION
IAV
D
Olfactory Epithelium
DESCARTES FETAL STOMACH LYMPHOID CELLS
DURANTE ADULT OLFACTORY NEUROEPITHELIUM GLOBOSE BASAL CELLS
DURANTE ADULT OLFACTORY NEUROEPITHELIUM MACROPHAGES
CUI DEVELOPING HEART C9 B T CELL
DURANTE ADULT OLFACTORY NEUROEPITHELIUM CD8 T CELLS
FAN EMBRYONIC CTX BIG GROUPS MICROGLIA
FAN EMBRYONIC CTX BRAIN B CELL
FAN EMBRYONIC CTX BRAIN EFFECTOR T CELL
DESCARTES FETAL EYE MICROGLIA
NES
INFECTION
IAV
E
Female 24dpi
Isg15
Fold Change vs Mock
Mock OB
IAV OB
SARS-CoV-2 OB
Mock OE
IAV OE
SARS-CoV-2 OE
F
Female 24dpi
Ccl5
Fold Change vs Mock
G
3dpi
Time to Finding Buried Food
Seconds
Mock
IAV
SARS-CoV-2
H
15 dpi
Time to Finding Buried Food
I
28dpi
Time to Finding Buried Food
J
3dpi Visible Food
Percentage Of Hamsters That Did Not Find Food
Seconds
K
15dpi Visible Food
L
28dpi Visible Food
Mock
IAV
SARS-CoV-2

fig. S6. SARS-CoV-2-infection is associated with olfactory epithelium inflammation and anosmia that resolves over time.

(A) In situ hybridization for SARS-CoV-2 spike protein was conducted on FFPE olfactory bulbs from SARS-CoV-2-infected hamsters at 3dpi and 31dpi. Spike (*S*) transcripts are visible as yellow puncta and 4',6-diamidino-2-phenylindole (DAPI) nuclear staining is visible in blue. Zoomed inset displays representative glomerular area of high positivity within each sample. The regions indicated with white dashed lines indicate glomeruli. Scale bars indicate 50μm. (MCL: Mitral Cell Layer; EPL: External Plexiform Layer; GL: Glomerular layer)

(B) FFPE olfactory bulbs from mock-, IAV-, or SARS-CoV-2-infected hamsters at 31dpi were processed by terminal deoxynucleotidyl transferase dUTP nick end labeling (TUNEL) staining to assess for apoptotic cells. The number of positive cells manually quantified in each olfactory bulb section is reported (n=4 bulbs assessed per infection group at 3dpi; n=4 SARS-CoV-2, n=4 IAV, and n=3 Mock treated bulbs assessed at 31dpi).

(C) Olfactory epithelium from SARS-CoV-2-, IAV-, and mock-infected hamsters was harvested at 31dpi and assessed using RNA-seq. Differential expression analysis was conducted with DESeq2 and analyzed by GSEA using curated gene ontology sets. Lollipop charts show significance of enrichment (-log10[FDR q-val]) (dot size) and NES for SARS-CoV-2 and IAV versus mock.

(**D**) GSEA was also performed on 31dpi olfactory epithelium differential expression data to assess for enrichment of cell-specific transcriptional signatures in SARS-CoV-2- and IAV-infected hamsters compared to mock. (CTX: Cortex)

(E and F) Olfactory bulbs and epithelium were harvested from an independent cohort of female hamsters at 24dpi and assessed for the presence of **(F)** *Isg15* and **(G)** *Ccl5* transcripts by qRT-PCR (n=2 per tissue per treatment group).

(G to L) The buried food-finding test was performed on hamsters at (**G)** 3, **(H)** 15, and **(I)** 28dpi (n=6 per treatment group per time point).. Seconds taken for each hamster to find buried food were plotted for as individual values for infection groups

tested at **(G)** 3dpi, **(H)** 15dpi, and **(I)** 28dpi. Following testing with time measured to discovery of buried food, the test was also repeated with food that was visible rather than buried. Kaplan-Meier curves demonstrate time to discovery of visible food for all infection groups at **(J)** 3dpi, **(K)** 15dpi, and **(L)** 28dpi.

Table S1: Metadata summary of olfactory bulb samples.

Tissue	Patient ID	Analysis Name	Sex	Age	Most Recent Positive COVID-19 Test (days prior to death)	COVID-19 Hospitalization (y/n)	Discharged from COVID-19 Hospitalization Prior to Death (y/n)	Cause of Death
OB	C1	Control	M	62	N/A	No	N/A	Acute cardiopulmonary event
OB	LP1	Long Post-COVID 1	F	72	128	Yes	Yes	Acute hypoxemic respiratory failure
OB	LP2	Long Post-COVID 2	M	65	189	Yes	Yes	Acute heart failure, chronic renal failure

Table S2: Metadata summary of olfactory epithelium samples.

Tissue	Patient ID	Analysis Name	Sex	Age	Most Recent Positive COVID-19 Test (days prior to death)	COVID-19 Hospitalization (y/n)	Discharged from COVID-19 Hospitalization Prior to Death (y/n)	Cause of Death
OE	C3	Control 1	M	74	N/A	No	N/A	Abdominal aortic aneurysm rupture
OE	C2	Control 2	M	57	N/A	No	N/A	Acute myocardial infarction
OE	C1	Control 3	M	62	N/A	No	N/A	Acute cardiopulmonary event
OE	LP3	Long Post-COVID 1	M	40	99	Yes	No	Cardiac arrest
OE	LP2	Long Post-COVID 2	M	65	189	Yes	Yes	Acute heart failure, chronic renal failure

Table S3: qRT-PCR primer sequences. N/A, not applicable.

Primer	Direction	Sequence
**SARS-CoV-2 sgN (TRS-L)**	N/A	CTCTTGTAGATCTGTTCTCTAAACGAAC
**SARS-CoV-2 sgN (TRS-N)**	N/A	GGTCCACCAAACGTAATGCG
**IAV NP**	Forward	GGCGAAGATGCAACAGCAGGTC
**IAV NP**	Reverse	GCACCAGACCTTCTGGGAAGTG
**ISG15**	Forward	TCTATGAGGTCCGGCTGACA
**ISG15**	Reverse	GCACTGGGGCTTTAGGTCAT
**MX2**	Forward	AGGCAGTGGTATTGTCACCAG
**MX2**	Reverse	ATCACTGATCCCCAGCCCTAT
**IRF7**	Forward	ATTTCGGTCGCAGGGATCTG
**IRF7**	Reverse	TGCAAGATAAAGCGTCCCGT
**CXCL10**	Forward	TCTGAGTGGGACTCAAGGAATC
**CXCL10**	Reverse	CCGGTCGGTCATCGATTTTG
**CCL5**	Forward	TCTCCACAGCTGTCCTCACT
**CCL5**	Reverse	TCCTTCGGGTGACAAAAACGA
**AIF-1/IBA-1**	Forward	GGGGAAAAGCCTTTGGACTG
**AIF-1/IBA-1**	Reverse	TCAAACTCCATGTACTTCTTCTTGA
