# Supplementary material for: SARS-CoV-2 infection in hamsters and humans results in lasting and unique systemic perturbations post recovery
Source: Sci Transl Med. 2022 Jun 7:eabq3059. doi: 10.1126/scitranslmed.abq3059 (PMC9210449; doi:10.1126/scitranslmed.abq3059)
Supplement: Supplementary file 2 — MDAR Reproducibility Checklist [file scitranslmed.abq3059_reproducibility_checklist.pdf]

Materials Design Analysis Reporting (MDAR)
Checklist for Authors

The MDAR framework establishes a minimum set of requirements in transparent reporting applicable to studies in the life sciences (see Statement of Task: doi:10.31222/osf.io/9sm4x.). The MDAR checklist is a tool for authors, editors, and others seeking to adopt the MDAR framework for transparent reporting in manuscripts and other outputs. Please refer to the MDAR Elaboration Document for additional context for the MDAR framework.

For all that apply, please note where in the manuscript the required information is provided.

Materials:

Newly created materials	indicate where provided: page no/section/legend)	n/a
The manuscript includes a dedicated "materials availability statement" providing transparent disclosure about availability of newly created materials including details on how materials can be accessed and describing any restrictions on access.	Our manuscript does not describe any newly generated materials but we did begin the Methods section with the requested statement.	

Antibodies	indicate where provided: page no/section/legend)	n/a
For commercial reagents, provide supplier name, catalogue number and RRID, if available.	All information about antibodies is included in the methods.	

DNA and RNA sequences	indicate where provided: page no/section/legend)	n/a
**Short novel DNA or RNA including primers, probes:** Sequences should be included or deposited in a public repository.	All RNA Seq data has been deposited on NCBI GEO using accession # GSE203001 (mentioned in methods / data accessibility section); primers used in this manuscript are reported as part of table S3	

Cell materials	indicate where provided: page no/section/legend	n/a
**Cell lines:** Provide species information, strain. Provide accession number in repository **OR** supplier name, catalog number, clone number, **OR** RRID.	All data generated derived directly from tissue samples of either hamsters or humans as indicated. The one exception are Vero-E6 cells which are used to plaque virus. These cells were purchased from ATCC (Cat. No. CRL-1586) (mentioned in methods section)	n/a
**Primary cultures:** Provide species, strain, sex of origin, genetic modification status.		n/a

Experimental animals	indicate where provided: page no/section/legend)	n/a
**Laboratory animals or Model organisms:** Provide species, strain, sex, age, genetic modification status. Provide accession number in repository **OR** supplier name, catalog number, clone number, **OR** RRID.	6–7 week-old male or female Golden Syrian hamsters (*Mesocricetus auratus*) were used where indicated and were obtained from Charles River Laboratories. (mentioned in methods section)	
**Animal observed in or captured from the field:** Provide species, sex, and age where possible.		n/a

Plants and microbes	indicate where provided: page no/section/legend)	n/a
**Plants:** provide species and strain, ecotype and cultivar where relevant, unique accession number if available, and source (including location for collected wild specimens).		n/a
**Microbes:** provide species and strain, unique accession number if available, and source.	SARS-CoV-2 isolate USA-WA1/2020 was propagated in Vero-E6 cells in DMEM supplemented with 2% FBS, 1mM HEPES and 1% penicillin/streptomycin. Virus stocks were filtered via centrifugation with Amicon Ultra-15 Centrifugal filter unit (Sigma) and sequenced to ensure maintenance of the furin cleavage site. All infections were performed with either passage 3 or 4 SARS-CoV-2. Influenza A virus H1N1 isolate A/California/04/2009 was propagated in MDCK cells in DMEM supplemented with 0.35% BSA, filtered and sequenced in a manner comparable to SARS-CoV-2 stocks.	

Human research participants	indicate where provided: page no/section/legend) or state if these demographics were not collected	n/a
If collected and within the bounds of privacy constraints report on age, sex and gender or ethnicity for all study participants.	Heart, lung, and kidney human samples were provided by the Weill Cornell Medicine Department of Pathology. The Tissue Procurement Facility operates under Institutional Review Board (IRB) approved protocol and follows guidelines set by Health Insurance Portability and Accountability Act (HIPAA). Experiments using samples from human subjects were conducted in accordance with local regulations and with the approval of the IRB at the Weill Cornell Medicine. The autopsy samples are considered human tissue research and were collected under IRB protocols 20-04021814 and 19-11021069. All autopsies have consent for research use from next of kin, and these studies were determined as exempt by IRB at Weill Cornell Medicine under those protocol numbers.<br><br>All autopsies are performed with consent of next of kin and permission for retention and research use of tissue. Autopsies were performed in a negative pressure room with protective equipment including N-95 masks; brain and bone were not obtained for safety reasons. All fresh tissues were procured prior to fixation and directly into Trizol for downstream RNA extraction. Tissues were collected from lung, kidney, and the heart as consent permitted. Post-mortem intervals ranged from less than 24 hours to 72 hours (with 2 exceptions - one at 4 and one at 7 days - but passing RNA quality metrics) with an average of 2.5 days. All deceased patient remains were refrigerated at 4˚C prior to autopsy performance.<br><br>Mentioned in methods and study design section	

Design:

Study protocol	indicate where provided: page no/section/legend)	n/a
If study protocol has been pre-registered, provide DOI. For clinical trials, provide the trial registration number **OR** cite DOI.		N/A

Laboratory protocol	indicate where provided: page no/section/legend)	n/a
Provide DOI **OR** other citation details if detailed step-by-step protocols are available.	Specific laboratory protocols are described in the method section. If full details are not available in these sections, they cite sources which provide access to full protocols.	

Experimental study design (statistics details)		
**For in vivo studies:** State whether and how the following have been done	**indicate where provided: page no/section/legend. If it could have been done, but was not, write not done**	**n/a**
Sample size determination	Sample size determination for specific experiments elaborated upon in study design section	
Randomisation	Hamsters were randomly assigned to infection groups at time of infection (mentioned in study design section)	
Blinding	All behavioral experiments and morphometric analyses were performed in a blinded manner (mentioned in study design section)	
Inclusion/exclusion criteria	Inclusion/exclusion and quality control criteria elaborated upon for individual experiments in methods section and figure legends	

Sample definition and in-laboratory replication	indicate where provided: page no/section/legend	n/a
State number of times the experiment was replicated in laboratory.	Study numbers are available in study design paragraph or in figure legends for the corresponding experiments. Key findings (most notably: olfactory bulb inflammatory phenotype) have been shown across multiple cohorts within our laboratory (in experiments reported here as well as non-reported experiments used for internal lab validation of results; experiments conducted by different individuals have displayed similar results).	
Define whether data describe technical or biological replicates.	Technical versus biological replication is expanded upon in methods and in figure legends for specific experiments. All statistics were generated using biological rather than technical replicates.	

Ethics	indicate where provided: page no/section/legend	n/a
**Studies involving human participants:** State details of authority granting ethics approval (IRB or equivalent committee(s), provide reference number for approval.	IRB and ethics committee reference numbers for use of human tissue samples are provided in the study design and methods sections.<br><br>For reference:<br><br>The study was approved by the ethics and Institutional Review Board of Columbia University Medical Center (IRB AAAT0689, AAAS7370).<br><br>The autopsy samples were considered human tissue research and were collected under IRB protocols 20-04021814 and 19-11021069. All autopsies had consent for research use from next of kin, and these studies were determined as exempt by IRB at Weill Cornell Medicine	

	under those protocol numbers.	
**Studies involving experimental animals:** State details of authority granting ethics approval (IRB or equivalent committee(s), provide reference number for approval.	All hamster experiments were covered under Mount Sinai IACUC committee under protocols: PROTO202000113 and IPROTO202100000053 This is mentioned in methods and study design sections	
**Studies involving specimen and field samples:** State if relevant permits obtained, provide details of authority approving study; if none were required, explain why.		n/a

Dual Use Research of Concern (DURC)	indicate where provided: page no/section/legend	n/a
If study is subject to dual use research of concern regulations, state the authority granting approval and reference number for the regulatory approval.		n/a

Analysis:

Attrition	indicate where provided: page no/section/legend	n/a
Describe whether exclusion criteria were preestablished. Report if sample or data points were omitted from analysis. If yes report if this was due to attrition or intentional exclusion and provide justification.	Specific criteria for inclusion into experiments is described in methods sections. Regarding omission of data points, n=1 statistical outlier was excluded from Marble Burying behavioral test. This outlier was identified via use of the iterative Grubbs outlier test (performed using Graphpad Prism).	

Statistics	indicate where provided: page no/section/legend	n/a
Describe statistical tests used and justify choice of tests.	Individual tests used are elaborated upon in methods section and in figure legends for respective experiments. Briefly, RNA-sequencing data was statistically analyzed using DESeq2 for differential expression. These data were further analyzed for enrichment of gene sets and transcriptional programs using Gene Set Enrichment Analysis, with -log10(p-value)/sign(log2FoldChange) used as the primary ranking factor. Additional data presented in the manuscripts as bar graphs was analyzed using ANOVA with post-hoc test appropriate for experiment (elaborated upon in figure legends and methods for respective experiments).	

Data availability	indicate where provided: page no/section/legend	n/a
For newly created and reused datasets, the manuscript includes a data availability statement that provides details for access or notes restrictions on access.	Provided within data accessibility section. Briefly, all RNA-sequencing data is available on NCBI GEO using accession # GSE203001. All additional numerical data generated in this experiment not dependent on these data is provided in supplemental data file (Data File S1)	
If newly created datasets are publicly available, provide accession number in repository **OR** DOI **OR** URL and licensing details where available.	Mentioned above and in data accessibility section.	
If reused data is publicly available provide accession number in repository **OR** DOI **OR** URL, **OR** citation.	Mentioned in study design section, methods section, and in data accessibility section.	

Code availability	indicate where provided: page no/section/legend	n/a
For all newly generated custom computer code/software/mathematical algorithm or re-used code essential for replicating the main findings of the study, the manuscript includes a data availability statement that provides details for access or notes restrictions.		n/a
If newly generated code is publicly available, provide accession number in repository, **OR** DOI **OR** URL and licensing details where available. State any restrictions on code availability or accessibility.		n/a
If reused code is publicly available provide accession number in repository **OR** DOI **OR** URL, **OR** citation.	Standard DESeq2 differential expression analysis code was reused in this study. Citations are provided for original sources of this code in the methods section addressing RNA-sequencing analysis.	

Reporting

MDAR framework recommends adoption of discipline-specific guidelines, established and endorsed through community initiatives. Journals have their own policy about requiring specific guidelines and recommendations to complement MDAR.

Adherence to community standards	indicate where provided: page no/section/legend	n/a
State if relevant guidelines (e.g., ICMJE, MIBBI, ARRIVE) have been followed, and whether a checklist (e.g., CONSORT, PRISMA, ARRIVE) is provided with the manuscript.		n/a
